# Signal Integration by the IκB Protein Pickle Shapes *Drosophila* Innate Host Defense

**DOI:** 10.1016/j.chom.2016.08.003

**Published:** 2016-09-14

**Authors:** Otto Morris, Xi Liu, Celia Domingues, Christopher Runchel, Andrea Chai, Shaherin Basith, Tencho Tenev, Haiyang Chen, Sangdun Choi, Giuseppa Pennetta, Nicolas Buchon, Pascal Meier

**Affiliations:** 1 The Breast Cancer Now Toby Robins Research Centre, Institute of Cancer Research, Mary-Jean Mitchell Green Building, Chester Beatty Laboratories, 237 Fulham Road, London SW3 6JB, UK; 2 Department of Entomology, Cornell University, 5124 Comstock Hall, 129 Garden Avenue, Ithaca, NY 14853, USA; 3 Euan MacDonald Centre for Motor Neuron Disease Research, Centre for Integrative Physiology, The University of Edinburgh, Hugh Robson Building, George Square, Edinburgh EH8 9XD, UK; 4 School of Life Sciences, Sun Yat-sen University, Guangzhou 510275, China; 5 Department of Molecular Science and Technology, Ajou University, Suwon 443-749, Korea; 6 National Leading Research Laboratory of Molecular Modeling & Drug Design, College of Pharmacy and Graduate School of Pharmaceutical Sciences, Ewha Womans University, Seoul 03760, Korea

**Keywords:** NF-κB, IκB, innate immunity, *Drosophila*, Imd

## Abstract

Pattern recognition receptors are activated following infection and trigger transcriptional programs important for host defense. Tight regulation of NF-κB activation is critical to avoid detrimental and misbalanced responses. We describe Pickle, a *Drosophila* nuclear IκB that integrates signaling inputs from both the Imd and Toll pathways by skewing the transcriptional output of the NF-κB dimer repertoire. Pickle interacts with the NF-κB protein Relish and the histone deacetylase dHDAC1, selectively repressing Relish homodimers while leaving other NF-κB dimer combinations unscathed. Pickle’s ability to selectively inhibit Relish homodimer activity contributes to proper host immunity and organismal health. Although loss of *pickle* results in hyper-induction of Relish target genes and improved host resistance to pathogenic bacteria in the short term, chronic inactivation of *pickle* causes loss of immune tolerance and shortened lifespan. Pickle therefore allows balanced immune responses that protect from pathogenic microbes while permitting the establishment of beneficial commensal host-microbe relationships.

## Highlights

• Pickle selectively inhibits NF-κB target genes that are driven by Relish homodimers
• Pickle is a nuclear member of the IκB protein family
• Loss of *pickle* causes hyper-activation of Relish-dependent target genes
• Loss of *pickle* enhances host resistance to bacteria but compromises lifespan

## Graphical Abstract

**Figure:**
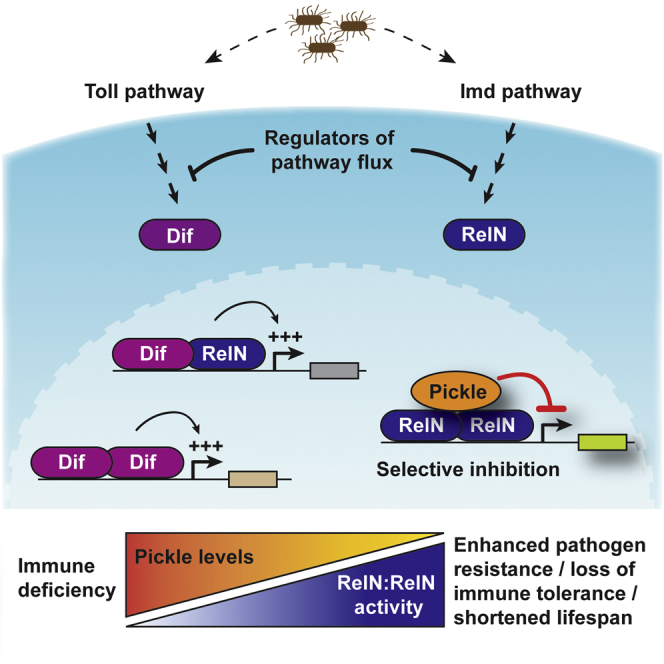

## Teaser

Tight regulation of NF-κB signaling is critical to avoid detrimental and misbalanced responses. Morris et al. identify an IκB protein in *Drosophila* that inhibits a selective subset of the NF-κB dimer repertoire, thereby ensuring an appropriate immune response to pathogens while preventing tissue damage and reduced lifespan.

## Introduction

Host defense against pathogen invasion relies on potent inflammatory responses that are controlled by the NF-κB family of transcription factors (Hayden and Ghosh, 2008). Activation of these transcription factors sets in motion a program aimed at clearing the pathogen. To restore homeostasis of the infected organ, such programs also induce modulators that, through negative feedback, regulate their temporal outputs to achieve balanced immune responses upon infection (Pasparakis, 2009).

NF-κB proteins share the presence of an N-terminal Rel homology domain (RHD), which is responsible for DNA binding as well as homo- and heterodimerization (Hayden and Ghosh, 2008). NF-κB proteins carry either an extended C-terminal stretch that contains multiple copies of ankyrin repeats (p105, p100, and *Drosophila* Relish) or a C-terminal transcription activation domain (c-Rel, RelB, RelA [p65], and the *Drosophila* Dorsal [dl] and Dif [Dorsal-related immune factor] protein) (Gilmore, 2006). NF-κB dimers bind to κB sites within the promoters and enhancers of target genes and regulate transcription through the recruitment of coactivators and corepressors (Hayden and Ghosh, 2008). The combinatorial diversity of NF-κB homo- and heterodimers contributes to the regulation of distinct, but overlapping, transcriptional programs (Smale, 2012).

The activity of NF-κB is regulated by interaction with inhibitory IκB proteins (Gilmore, 2006). The IκB family proteins include, at least, eight dedicated IκB proteins: IκBα, IκBβ, IκBγ, IκBε, IκBζ, IκBNS, Bcl-3, and *Drosophila* Cactus. All IκB proteins harbor multiple ankyrin repeat regions (ARRs) through which IκBs bind to the RHDs of NF-κB dimers and regulate their transcriptional response. Generally, individual IκBs associate preferentially with a particular set of NF-κB dimers (Gilmore, 2006). Studying the function, mechanism of activation, and regulation of these factors is crucial for understanding host responses to microbial infections, immunological memory, and commensal-host interactions.

*Drosophila* can engage two pathways to activate NF-κB: the Toll pathway is activated primarily by fungal and Gram-positive infections, while the *Immune deficiency* (*Imd*) pathway responds mainly to Gram-negative infections (Buchon et al., 2014, Lemaitre et al., 1995, Lemaitre et al., 1996).

Toll activation is triggered by Lys-type peptidoglycans (PGNs) as well certain bacterial virulence factors and components of fungal cell walls (El Chamy et al., 2008, Gottar et al., 2006, Michel et al., 2001). The Toll pathway initiates via an extracellular proteolytic cascade that culminates in the cleavage and activation of Spatze (Spz), which binds to the transmembrane Toll receptor and initiates an intracellular signaling cascade that results in the phosphorylation-dependent degradation of the IκB protein Cactus (Ganesan et al., 2011). This enables nuclear translocation of the NF-κB transcription factors Dif and dl (Lemaitre et al., 1996, Manfruelli et al., 1999, Rutschmann et al., 2002). Of these NF-κB proteins, Dif is the predominant transactivator in the antifungal and anti-Gram-positive bacterial defense in adults (Lemaitre et al., 1996, Manfruelli et al., 1999, Meng et al., 1999, Rutschmann et al., 2000a). Dorsal can substitute for Dif in larvae (Manfruelli et al., 1999, Rutschmann et al., 2000b).

The Imd pathway is activated by Gram-negative bacteria via two DAP-type PGN recognition receptors, plasma-membrane PGRP-LC and cytosolic PGRP-LE (Buchon et al., 2014). Binding of PGN to the receptors results in recruitment of an Ub-dependent signaling complex consisting of Imd, dFadd, and the caspase-8 homolog Dredd (Ganesan et al., 2011). Dredd is activated in an Ub-dependent manner with the help of the E3-ligase inhibitor of apoptosis 2 (Diap2) (Kleino et al., 2005, Leulier et al., 2006, Meinander et al., 2012). Once active, Dredd cleaves off an inhibitory C-terminal ankyrin repeat of Relish, allowing translocation of the active RHD-containing N-terminal portion (RelN) to the nucleus, where it can act to induce activation of Relish-dependent target genes (Ganesan et al., 2011).

Activation of Toll and Imd pathways induces the expression of distinct but overlapping groups of NF-κB responsive antimicrobial peptide (AMP) genes, which are important for fending off invading microorganisms (Buchon et al., 2014). Because dl, Dif, and RelN readily form homo- as well as heterodimers, the transcriptional output of NF-κB can vary depending on dimer compositions and co-factor association (Bonnay et al., 2014, Busse et al., 2007, Goto et al., 2008, Han and Ip, 1999, Tanji et al., 2007, Tanji et al., 2010). How organisms are able to detect the presence of pathogens, and in response trigger balanced expression of innate defense genes, is a major question. It is clear that the expression repertoire and duration of immune defense genes must be tightly balanced to effectively clear pathogens while avoiding deleterious immune activation and tissue damage. Whereas pathogens frequently trigger multiple pattern recognition receptors, it remains unclear how these signals are integrated into an appropriate defense response to clear the pathogen. Here we report the identification and characterization of a *Drosophila* member of the IκB superfamily, which we term Pickle.

## Results

### Pickle Negatively Regulates the NF-κB Transcription Factor Relish

**Figure 1:**
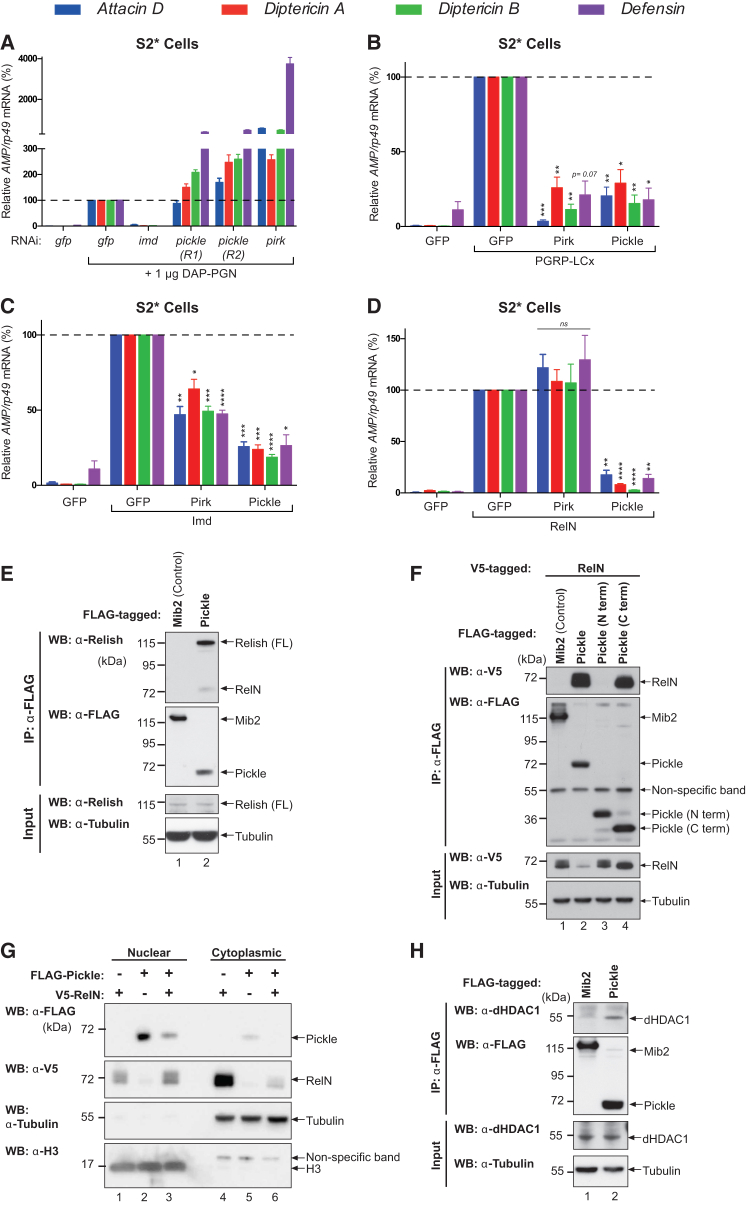
Pickle Negatively Regulates Relish (A–D) qRT-PCR analysis of mRNA from S2^∗^ cells. (A) Relative *AMP* mRNA levels before and after 4 hr of treatment with DAP-PGN in the presence of the indicated double-stranded RNAs (dsRNAs). *R1* and *R2* depict dsRNAs targeting two non-overlapping regions (R) of *pickle*. (B–D) Relative *AMP* mRNA levels of S2^∗^ cells transiently transfected with the indicated constructs. V5-tagged RelN was used. (E and F) FLAG immunoprecipitation of the indicated proteins was performed in S2^∗^ cells, and Relish binding was assessed via western blot. (G) Nuclear and cytoplasmic extracts of S2^∗^ cells transfected with the indicated proteins were analyzed by western blot. Equal total protein was loaded for both extracts. (H) FLAG-tagged Pickle and FLAG-tagged Mib2 (control) were expressed in S2^∗^ cells. FLAG immunoprecipitation was performed and binding of endogenous dHDAC1 to Pickle, or Mib2, was assessed via western blot. Histograms express results as percentage of a control sample (marked with dotted line). Unless otherwise indicated, p values were calculated from control using an unpaired Student’s t test. Results are representative of three (B–H) or two (A) biological repeats. Mean ± SEM of biological (B–D) or experimental (A) repeats. ^∗^p ≤ 0.05, ^∗∗^p ≤ 0.01, ^∗∗∗^p ≤ 0.001, and ^∗∗∗∗^p ≤ 0.0001. See also Figure S1.

To identify regulators of NF-κB signaling, we performed an in vitro RNAi mini-screen of proteins that interact with the *Drosophila* NF-κB protein Relish (Guruharsha et al., 2011, Rhee et al., 2014). This identified CG5118 as a putative negative regulator of Relish (Figure 1). In S2^∗^ cells, knockdown of CG5118, subsequently referred to as Pickle, caused hyperinduction of Imd-dependent AMP (*AMP*) genes following treatment with PGN from Gram-negative bacteria (Figures 1A, S1A, and S1B). Conversely, overexpression of Pickle strongly suppressed PGRP-LCx-, Imd-, and RelN-mediated induction of *AMP*s (Figures 1B–1D). This suggests that Pickle regulates the Imd pathway at the level of RelN. Accordingly, Pickle had no effect on Relish processing upon immune activation (Figure S1C). Whereas Pickle inhibited both Imd- and RelN-mediated production of *AMP*s, Pirk suppressed only PGRP-LCx- and Imd-induced activation of *AMP* genes.

The observation that Pickle suppresses RelN-driven induction of *AMP*s strongly suggests that Pickle directly regulates active, processed Relish. Consistently, we found that Pickle readily bound to the RelN portion of Relish (Figures 1E, 1F, and S1D), which is in agreement with previous proteomic-based studies (Guruharsha et al., 2011, Rhee et al., 2014). Detailed interaction analysis revealed that Pickle homo-oligomerizes (Figure S1E) and that the C-terminal half (aa 277–525) of Pickle was necessary and sufficient for RelN binding (Figure 1F). Although Pickle efficiently bound to Relish, it did not interact with other members of the *Drosophila* NF-κB family, such as dl and Dif (Figure S1D). Subcellular fractionation revealed that FLAG-tagged Pickle predominantly resides in the nuclear fraction (Figure 1G). Intriguingly, expression of Pickle appeared to sequester RelN in the nucleus, as significantly less RelN was present in the cytoplasmic fraction following co-expression with Pickle (Figure 1G).

The histone deacetylase dHDAC1 (also referred to as Rpd3) reportedly negatively regulates the transactivation of Relish (Kim et al., 2005, Kim et al., 2007), even though dHDAC1 does not directly bind to Relish (Kim et al., 2007). We therefore tested whether Pickle interacts with dHDAC1. We found that Pickle selectively co-purified endogenous dHDAC1 from cellular extracts (Figure 1H). Together, our data suggest that Pickle is a negative regulator of the Imd pathway that binds and inhibits the activity of the Relish, possibly via dHDAC1 recruitment.

### Pickle Is a Member of the IκB Superfamily of Proteins

**Figure 2:**
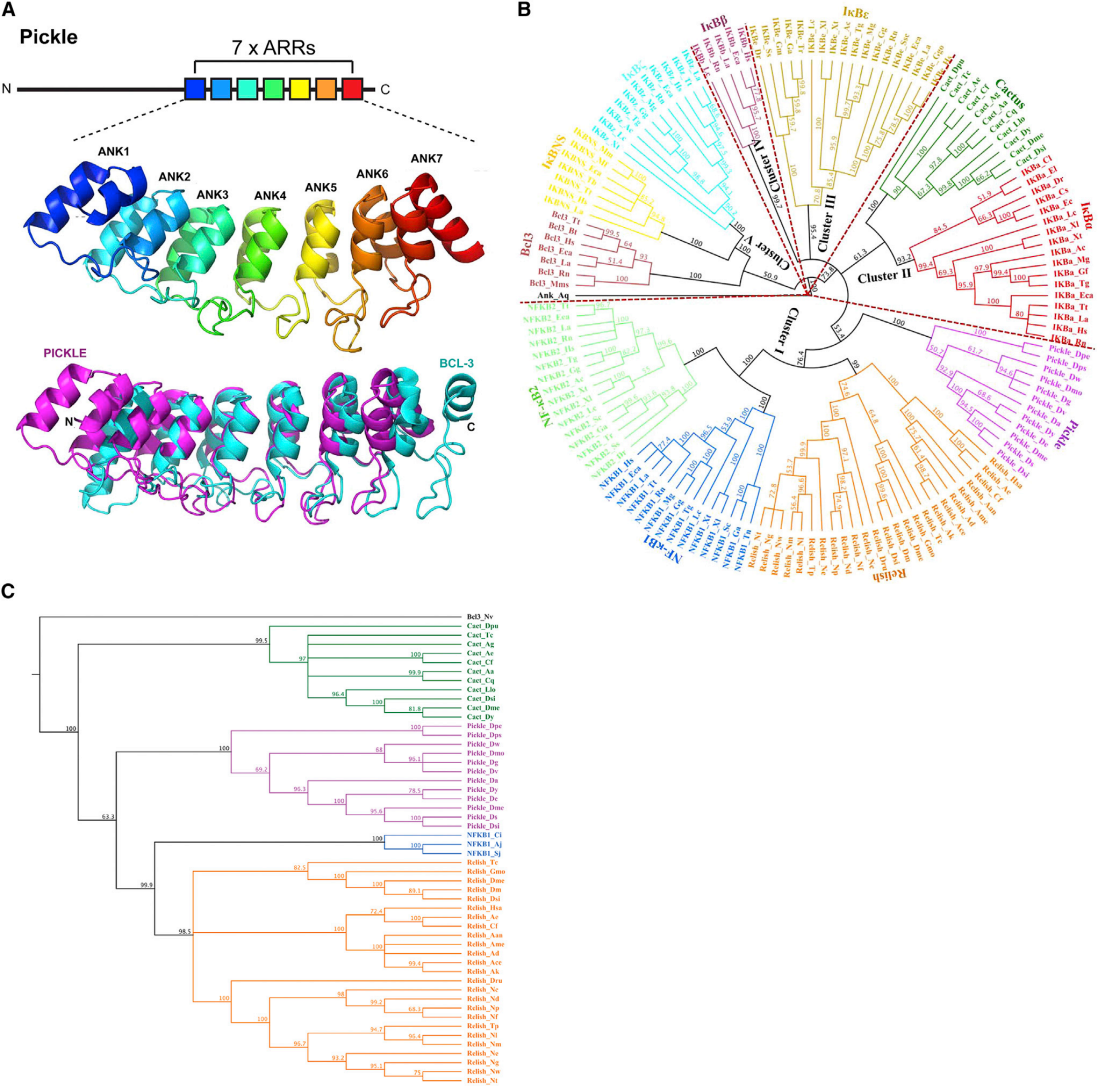
Phylogenetic Relationship of Pickle with Other IκB Family Members (A) Schematic representation of Pickle (top) and its predicted 3D structure (middle). The predicted structure of the seven ARRs of Pickle (magenta) was superimposed onto the structure of Bcl-3 (PDB: 1K1A; cyan) (bottom). (B) Phylogenetic analysis of IκB proteins. The sponge *Amphimedon queenslandica* was considered as an out-group. Bootstrap values > 50% have been provided. Members: IκBα (red), IκBβ (wine), IκBε (tan), Bcl-3 (brown), IκBNS (yellow), IκBζ (cyan), Cactus (dark green), Relish (orange), NF-κB1 (blue), NF-κB2 (light green), and Pickle (magenta). (C) Phylogenetic relationship of Pickle with IκB family members present in invertebrates only using neighbor-joining method. Bcl-3 from *Nematostella vectensis* was considered as an outgroup (shown in black). Bootstrap scores > 60% have been provided. Members: Cactus (dark green), Relish (orange), NF-κB1 (blue), and Pickle (magenta). See Table S1 for details. See also Figure S2.

All currently known IκB proteins from vertebrates and invertebrates carry C-terminal ARRs with which they bind to the RHDs of NF-κB proteins (Hayden and Ghosh, 2008). Using sequence analysis and structural prediction algorithms, we identified seven ARRs within the C-terminal portion of Pickle (Figures 2A and S2), the portion that is necessary and sufficient for Relish binding. The N-terminal portion of Pickle did not harbor any recognizable motifs or domains. Because Pickle selectively binds to the RHD of Relish via its C-terminal ARRs and inhibits Relish activity, Pickle fulfils all functional and structural criteria of IκB proteins.

Phylogenetic analysis of Pickle with all currently known IκBs revealed that Pickle, along with its orthologs, is part of a clade of the IκB phylogenetic tree. IκB phylogenetic rooted tree reconstruction identified five major clades among the IκB proteins (Figure 2B). These major clades include (1) Pickle and Relish with NF-κB1 and NF-κB2 (53.4% bootstrap value), (2) Cactus with IκBα (61.3% bootstrap value), (3) IκBε (95.4% bootstrap value), (4) IκBβ (99.7% bootstrap value), and (5) Bcl-3 with IκBζ and IκBNS (nuclear IκB proteins; 50.9% bootstrap value). The tree organization was validated using rooted and unrooted phylogenetic trees of invertebrate IkBs (Figure 2C). Pickle clustered along with Relish in both whole IκB and invertebrate-specific phylogenetic trees, with a bootstrap support of 100%. Our distance analysis demonstrates that *pickle* represents the direct arthropod homolog of the *relish* gene, albeit lacking a RHD in the N terminus and a PEST domain in its C terminus. Taken together, our functional, phylogenetic, and sequence analysis identifies Pickle, and its orthologs, as a member of the IκB superfamily.

### Loss of *pickle* Results in Hyper-Activation of Relish Target Genes upon Infection

**Figure 3:**
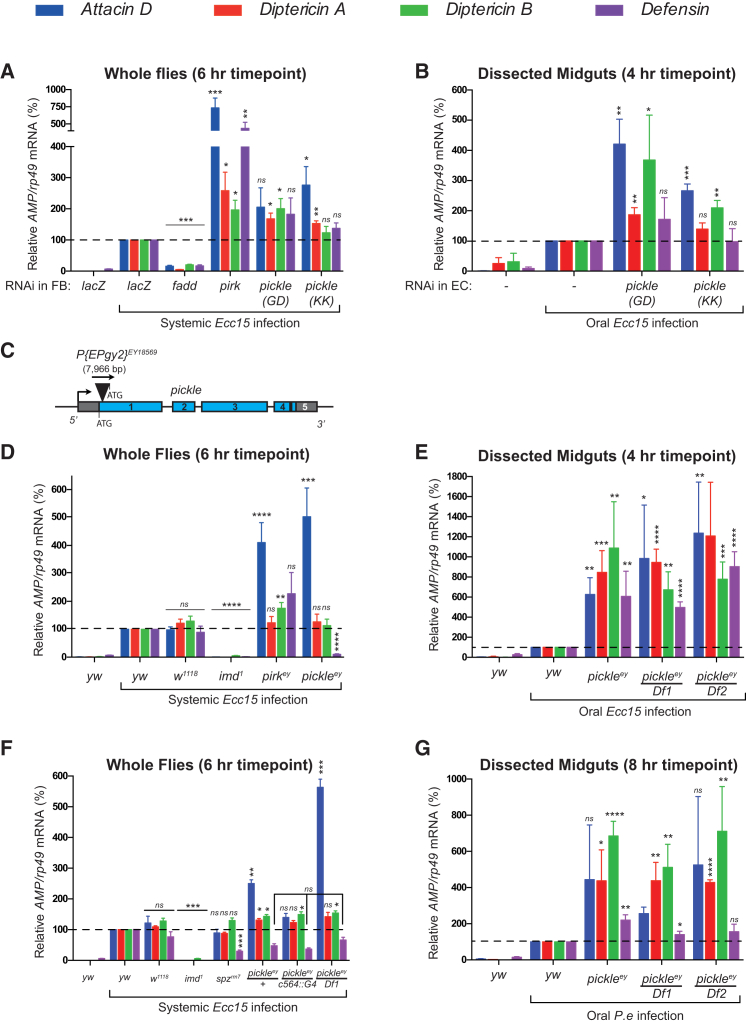
Loss of *pickle* Causes Hyperinduction of AMPs following Infection with Gram-Negative Bacteria (A, B, and D–G) qRT-PCR analysis of the indicated genotypes. (A) Relative *AMP* mRNA levels from whole flies before and after 6 hr of infection with *Ecc15* (∼2,000 CFU). RNAi of the indicated target genes was driven in the fat body (FB) using *c564::Gal4*. *pickle* (*GD*) and *pickle* (*KK*) refer to two transgenic lines encoding dsRNAs that target non-overlapping regions of *pickle*. (B) Relative *AMP* mRNA levels of dissected midguts before and after 4 hr of oral infection with *Ecc15*. RNAi knockdown was restricted to enterocytes (EC) using *myo::Gal4*. (C) Schematic representation of the *pickle* gene depicting the insertion site of the transposon *P[EPgy2]*^EY18569^. (D and E) The indicated flies were treated as in (A) and (B), respectively. *Df1* refers to the *Df(2L)Exel7006* deletion. *Df2* refers to *Df(2L)BSC481*. (F) The indicated flies were analyzed as in (A). (G) Relative *AMP* mRNA levels from dissected midguts before and after 8 hr of infection with *P.e* oral infection. Histograms express results as percentage of a control sample (marked with dotted line). Unless otherwise indicated, p values were calculated from control using an unpaired Student’s t test. Results are representative of at least three biological repeats (mean ± SEM). ^∗^p ≤ 0.05, ^∗∗^p ≤ 0.01, ^∗∗∗^p ≤ 0.001, and ^∗∗∗∗^p ≤ 0.0001. See also Figure S3.

Next we investigated the role of Pickle in regulating *Drosophila* innate immune responses. Septic injury with the Gram-negative bacteria *Erwinia carotovora carotovora 15* (*Ecc15*) resulted in hyper-activation of Imd signaling in flies in which *pickle* was knocked down in the fat body (Figures 3A and S3A). Although knockdown of *pickle* resulted in hyper-activation of Relish target genes, *pickle* inactivation did not affect Dif-mediated induction of *Drosomycin* following activation of the Toll pathway via septic injury with the Gram-positive, Lys-type PGN containing bacteria *Micrococcus luteus* (*M.lut*) (Figures S3C and S3D). Pickle, therefore, selectively modulates Imd signaling.

*pickle* also controlled the Imd pathway in the fly midgut following oral infection. Accordingly, feeding Gram-negative *Ecc15* or *Pseudomonas entomophila* (*P.e*) caused upregulation of multiple Relish target genes in dissected midguts (Figures 3B and S3E). Compared with control flies, induction of Relish-dependent genes was significantly greater in flies with enterocyte-specific knockdown of *pickle* (Figures 3B, S3A, S3B, and S3E). *pickle*^P[EPgy2]EY18569^ null mutant flies (hereafter referred to as *pickle*^ey^), which carry a transposon inserted 24 bp downstream of the translational start site of *pickle* (Figures 3C and S3G), also hyper-activated Relish target genes following systemic infection with *Ecc15* (Figure 3D). Likewise, oral infection with *Ecc15* or *P.e* similarly caused a dramatic over-production of Relish-dependent target genes (Figures 3E and 3G). Essentially the same results were obtained using either homozygous *pickle*^ey^ mutant animals or trans-heterozygous *pickle*^ey/Df1^ or *pickle*^ey/Df2^ flies that carry deletions of the *pickle* locus (*Df1*: *Df[2L]Exel7006*; *Df2*: *Df[2L]BSC481*) (Figures 3D, 3E, 3G, and S3F). Of note, following systemic infection, *Defensin* induction was strongly reduced in homozygous *pickle*^ey^ flies when compared to wild-type (WT) animals (*yw* and *w*^1118^). This effect is due to a background mutation in *pickle*^ey^ flies because the reduced *Defensin* levels did not complement when *pickle*^ey^ was placed trans-heterozygous over *pickle*-uncovering deficiency alleles (Figures 3F and S3F). This background effect only affects the expression of *Defensin*, not other *AMPs*, and was observed following only systemic, not oral, infection (Figures 3D–3G and S3F). This is evident as oral infection with *Ecc15* or *P.e* caused elevated *Defensin* levels in homozygous *pickle*^ey^ flies that were comparable with those of *pickle*^ey/Df1^ and *pickle*^ey/Df2^. To circumvent this background effect, all subsequent systemic infection experiments were conducted using *pickle*^ey/Df1^, *pickle*^ey/+^, and *pickle*^ey/c564^ genotypes, allowing the comparison of flies with zero (*pickle*^ey/Df1^) or one WT copy (*pickle*^ey/+^) and one WT copy with one allele re-expressing Pickle in the fat body (*pickle*^ey/c564^). Together, our data indicate that *pickle* negatively regulates the Imd pathway, upon both systemic and oral infections.

Although loss of *pickle* resulted in hyper-activation of Relish target genes following systemic infection, fat body-specific and *P[EPgy2]* transposon-mediated re-expression of *pickle* fully rescued *AMP* expression to normal levels (Figure 3F). The *P[EPgy2]* transposon in the *pickle* locus (*EY18569*) carries an upstream activating sequence element that permits GAL4-mediated re-expression of *pickle* commencing from an ATG at the end of the *P[EPgy2]* transposon (Bellen et al., 2004). *P[EPgy2]* transposon-mediated re-expression generates Pickle lacking the eight N-terminal residues (Figure S3G). Because re-expression of Pickle rescues hyper-activation of *AMPs* in *pickle*^ey^ flies, our data indicate that the *pickle*^ey^ phenotype is indeed due to loss of *pickle*.

### *pickle* Suppresses Spontaneous Induction of Relish-Dependent Target Genes in the Absence of Infection and Maintains Fly Lifespan

**Figure 4:**
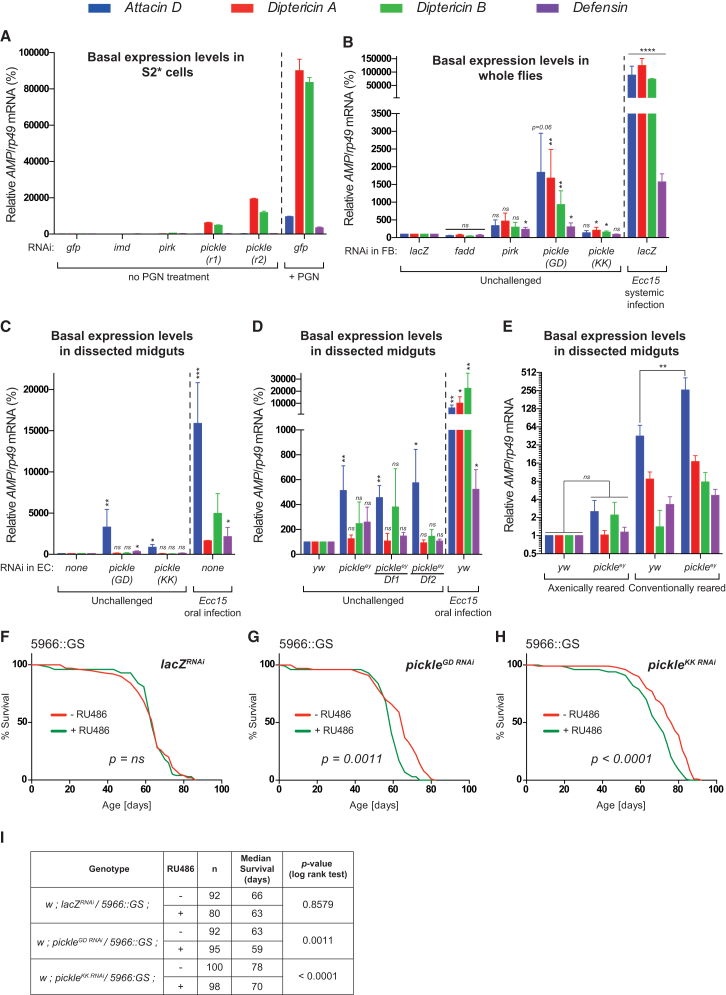
*pickle* Suppresses Spontaneous Induction of Relish-Dependent Target Genes in the Absence of Infection and Maintains Lifespan (A–E) qRT-PCR analysis of mRNAs of the indicated samples. (A) Relative *AMP* mRNA levels from unchallenged S2^∗^ cells following RNAi of the indicated genes. S2^∗^ cells treated for 4 hr with DAP-PGN are shown as reference point. (B) Relative *AMP* mRNA levels from unchallenged whole flies. RNAi knockdown was restricted to fat body (FB) cells using *c564::Gal4*. Relative *AMP* mRNA levels of control flies injected with *Ecc15* (2,000 CFU) (6 hr) served as reference point. (C) Relative *AMP* mRNA levels of dissected midguts from unchallenged female flies. RNAi knockdown was restricted to enterocytes (EC) using *myo::Gal4*. (D) Analysis of flies with the indicated genotypes was conducted as in (C). (E) Relative *AMP* mRNA levels of dissected midguts from unchallenged female flies reared under conventional or axenic conditions. (F–H) Lifespan experiments using the geneswitch system. Knockdown was restricted to enteroblasts (EBs)/enterocytes (ECs) using the geneswitch driver *5966:GS*. (I) Statistical summary of experiments shown in (F–H). Histograms express results as percentage of a control sample (marked with dotted line). Unless otherwise indicated, p values were calculated from control using an unpaired Student’s t test. Results are representative of three (B–E) or two biological repeats (A). Mean ± SEM of biological (B–E) or experimental (A) repetitions. ^∗^p ≤ 0.05, ^∗∗^p ≤ 0.01, ^∗∗∗^p ≤ 0.001, and ^∗∗∗∗^p ≤ 0.0001.

For a host to tolerate a certain amount of resident bacteria, it is critical that the activation threshold of the immune response be tightly regulated (Buchon et al., 2014). Because *pickle* is a selective negative regulator of Relish, we investigated whether *pickle* contributes to the activation threshold of Relish-dependent target genes by suppressing Relish activity. Using the sterile environment of S2^∗^ cells, we found that mere knockdown of *pickle* led to a dramatic induction (>5,000-fold) of the basal levels of *Diptericin A* (*DiptA*) and *Diptericin B* (*DiptB*) (Figure 4A). Likewise, tissue-specific knockdown of *pickle* in the gut (enterocytes) or fat body led to a marked increase in the basal levels of *AMP* gene expression in unchallenged flies (Figures 4B and 4C). Transcript levels of *AMP* genes were also significantly elevated in dissected midguts of unchallenged *pickle*^ey^ and trans-heterozygous *pickle*^ey/Df1^ and *pickle*^ey/Df2^ animals (Figure 4D). However, unlike in S2^∗^ cells, the elevated expression of *AMPs* in midguts of *pickle*^ey^ flies was dependent on the presence of commensal bacteria, as this phenotype was lost when flies were reared under sterile conditions (Figure 4E). These data suggest that Pickle contributes to immune tolerance in the gut, preventing aberrant Relish-activity in response to gut microbiota. The difference between S2^∗^ cells and cells of the midgut may reflect cell- and tissue-type dependent differences.

Previous work indicated that chronic hyper-activation of Imd signaling in the gut reduces lifespan (Guo et al., 2014, Paredes et al., 2011). To test whether loss of *pickle* impacts on lifespan, we made use of the GeneSwitch system (Mathur et al., 2010), which negates genetic background effects (He and Jasper, 2014). Consistent with the notion that gut-specific knockdown of *pickle* results in hyper-activation of Imd signaling, we found that long-term, GeneSwitch-mediated depletion of *pickle* in enteroblasts and enterocytes caused a significant reduction in lifespan (Figures 4G–4I). Under the same conditions, GeneSwitch-mediated depletion of *lacZ* had no effect (Figures 4F and 4I). Together these data demonstrate that depletion of *pickle* results in hyper-activation of Imd signaling in the gut, which, similar to the loss of other Imd pathway negative regulators (Paredes et al., 2011), may compromise lifespan.

### *pickle* Is Induced in Response to Commensal and Infectious Bacteria

**Figure 5:**
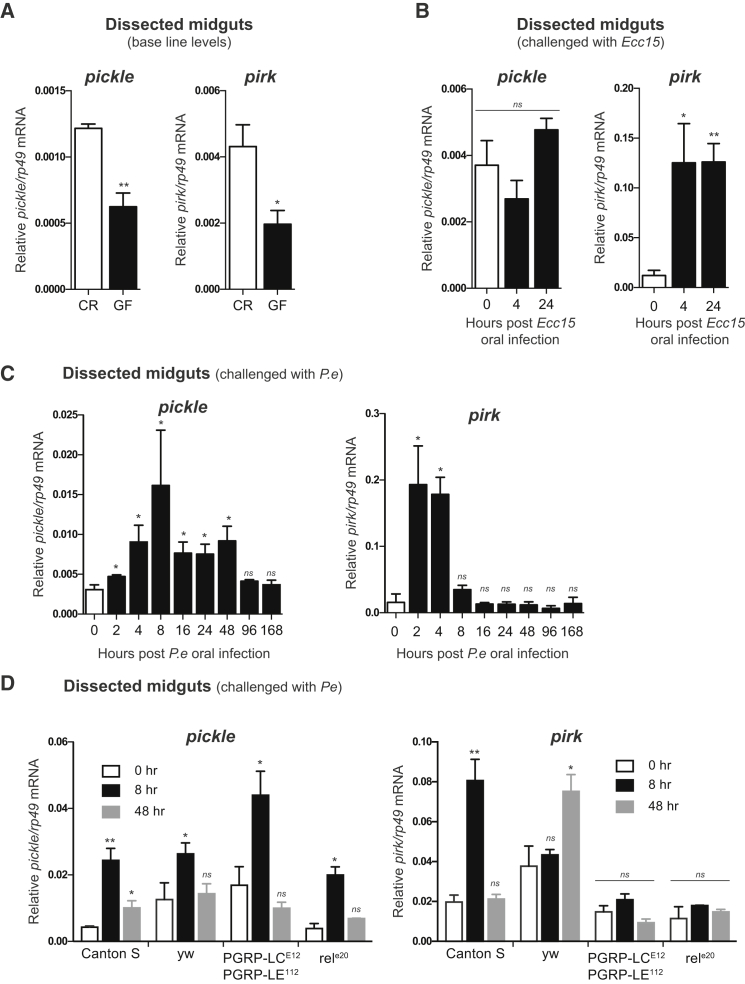
*pickle* Expression Is Induced in Response to Commensal and Infectious Bacteria (A–D) qRT-PCR analysis of *pickle* or *pirk* transcript levels. Unless otherwise stated, results are from dissected midguts of *Canton S* flies. (A) Relative mRNA levels of *pickle* and *pirk* in CR and GF flies. (B) Relative mRNA levels of *pickle* and *pirk* following oral infection with *Ecc15* or (C) *P.e*. (D) Relative mRNA levels of *pickle* and *pirk* in dissected midguts of the indicated genotypes following oral infection with *P.e*. p values were calculated from respective control (white bars) using an unpaired Student’s t test. Results are representative of three biological repetitions (mean ± SEM). ^∗^p ≤ 0.05, ^∗∗^p ≤ 0.01, ^∗∗∗^p ≤ 0.001, and ^∗∗∗∗^p ≤ 0.0001. See also Figure S4.

Expression of several negative regulators of the Imd pathway, such as *pirk* and *PGRP-LB*, are regulated by Relish, allowing negative-feedback control of Imd signaling (Aggarwal et al., 2008, Kleino et al., 2008, Lhocine et al., 2008, Zaidman-Rémy et al., 2006). We found that *pickle* levels were significantly higher in midguts of conventionally reared (CR) animals than in germ free (GF) counterparts (Figure 5A). This indicates that *pickle* expression in the midgut is influenced by the presence of commensal bacteria, an observation that is consistent with a recent micro-array study (Broderick et al., 2014). Following oral infection, induction of *pickle* varied depending on the type of Gram-negative bacteria. Whereas oral infection with *Ecc15* did not induce *pickle* expression (Figure 5B), exposure to the entomopathogenic bacteria *P.e* caused a significant increase in *pickle* expression (Figure 5C). A similar bacteria-specific induction of *pickle* was also noted previously (Buchon et al., 2009a, Chakrabarti et al., 2012). Unlike *pickle*, expression of *pirk* increased in response to both these Gram-negative bacteria (Figures 5B–5D). Consistent with the notion that *pickle* and *pirk* are regulated differently, we found that exposure to *P.e* induced *pickle* independently of PGRP-LC/LE, Imd, and Relish (Figure 5D). Upon systemic infection, the induction of *pickle* is relatively modestly (<2 times) (Figure S4A), which is in agreement with previous micro-array studies (De Gregorio et al., 2002, Irving et al., 2001). This was unlike *pirk*, which was strongly upregulated in an Imd-dependent manner upon systemic infection (Figure S4B). Although the pathway or pathways that regulate *pickle* expression remain to be identified, *pickle* expression in the midgut appeared not to be induced by tissue damage per se (Figures S4C and S4D). Together, our data demonstrate that *pickle* is induced, albeit moderately, in response to commensal microbiota, and infection with certain types of bacteria.

### Pickle Selectively Inhibits RelN Homodimers

**Figure 6:**
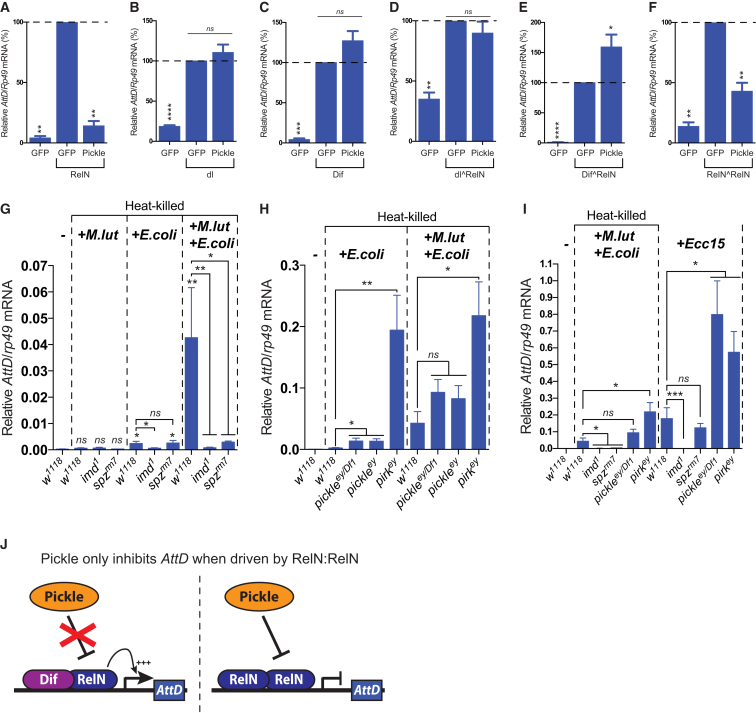
Pickle Selectively Inhibits RelN Hmodimers (A–F) Relative *AttD* mRNA levels of S2^∗^ cells transiently transfected with plasmids expressing the indicated proteins. All proteins are FLAG-tagged at their N termini. Histograms depict mean ± SEM of three biological repeats. Results are expressed as percentage of induced GFP control samples in each experiment, and statistical significance is measured from these using an unpaired Student’s t test. (G–I) Relative *AttD levels* mRNAs from unchallenged flies or flies injected with the indicated hk or live (*Ecc15*, 2,000 CFU) bacteria (6 hr). Unless otherwise indicated, statistical significance was measured from unchallenged *w*^1118^ flies using an unpaired Student’s t test. (J) Model depicting Pickle-mediated regulation of RelN. ^∗^p ≤ 0.05, ^∗∗^p ≤ 0.01, ^∗∗∗^p ≤ 0.001, and ^∗∗∗∗^p ≤ 0.0001. See Figure S5.

The RHD of NF-κB proteins mediates DNA binding as well as homo- and heterodimerization (Hayden and Ghosh, 2008). In *Drosophila*, concomitant activation of the Toll and Imd pathways reportedly drives the formation of a complex network of Dif, dl, and Relish homo- and heterodimers (Tanji et al., 2010). Different dimer combinations are thought to activate overlapping transcriptional programs that vary in intensity, duration, and target genes (Smale, 2012). Because Pickle selectively binds to RelN (Figure 1), we tested the ability of Pickle to regulate various NF-κB homo- and heterodimer combinations. Whereas expression of Pickle strongly suppressed the transactivation ability of RelN as well as linked RelNˆRelN homodimers (Figures 6A, 6F, S5A, and S5F; the caret represents the flexible peptide linker), Pickle failed to inhibit Dif, dl, and linked dlˆRelN or DifˆRelN dimer combinations (Figures 6B–6E and S5B–S5E). Of note, the ability of Pickle to repress induction of *AttD* and *AttA* was irrespective of the level of induction (Figures 1D and S5G–S5J). Intriguingly, the inability of Pickle to suppress linked DifˆRelN and dlˆRelN was not due to lack of Pickle-binding, as Pickle readily co-purified DifˆRelN and dlˆRelN from cellular extracts (Figure S5K). This suggests that Pickle requires two RelN moieties to inhibit transactivation.

Next, we investigated the impact of Pickle when both the Imd and Toll pathways are simultaneously activated in vivo. To that end, we used injection of heat-killed (hk) *E. coli* (*E.coli*) and *M.lut* and examined gene expression after 6 hr. Heat-killed bacteria were used to avoid any complication due to different bacterial growth rates. Interestingly, we found that loss of *pickle* (*pickle*^ey^ and *pickle*^ey/Df1^) hyper-activated *AttD* only when *AttD* was driven by RelN-only, such as following injection with *E.coli* (hk) (Figures 6G–6J, S5L, and S5M). In contrast, loss of *pickle* had no effect on *AttD* expression following co-injection of *E.coli* (hk) + *M.lut* (hk) (Figure 6H), a condition that induces *AttD* expression in an Imd- and Toll-dependent manner. This is entirely consistent with the notion that Pickle selectively inhibits target gene induction when such genes are exclusively driven by RelN. Of note, the overall level of *AttD* induction did not influence the ability of *pickle* to regulate RelN-driven expression of *AttD*. This is evident as injection of live *Ecc15*, which drives *AttD* induction in a purely Imd-dependent manner, triggered the strongest upregulation of *AttD* (Figure 6I). Nevertheless, loss of *pickle* caused significant hyper-activation of *AttD*. Overall, our data strongly suggest that Pickle selectively inhibits RelN homodimers, while leaving Dif:RelN heterodimers unscathed (Figure 6J). Of note, at present we cannot rule out the possibility that synergistic induction of *AMPs* is mediated by cooperating homodimers (Figure S5N), instead of heterodimers. Regardless of whether the *Drosophila* NF-κB proteins can act as either self-contained heterodimers or cooperating homodimers, our data clearly demonstrate that Pickle only affects target gene expression when such genes are driven exclusively by RelN-only. As such, these data are entirely consistent with our observations using compound NF-κB dimers in S2^∗^ cells.

### *pickle* Alters Host Resistance following Infection with Pathogenic Bacteria

**Figure 7:**
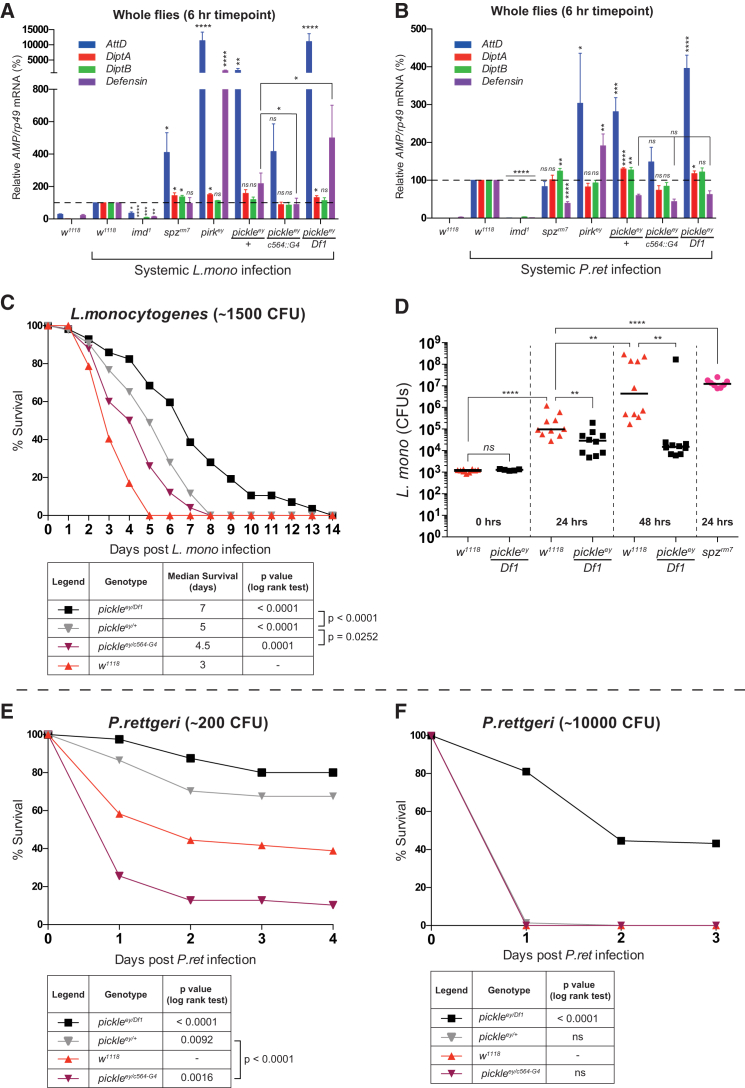
Loss of *pickle* Improves Host Resistance to Pathogenic Bacteria (A and B) qRT-PCR analysis of *AMP* mRNAs of the indicated flies before and 6 hr post-systemic infection with (A) *L.mono* (∼1,500 CFU) and (B) *P.ret* (∼10,000 CFU). Results are expressed as percentage of the induced levels of control flies (*w*^1118^) in each experiment (marked with a dotted line), and statistical significance was measured from these using an unpaired, two-tailed Student’s t test. Histograms depict mean ± SEM of three biological repetitions. (C) Kaplan-Meier plot showing the survival of female flies injected with *L.mono* (∼1,500 CFU). Statistical significance between the survival of infected flies and WT controls (*w*^1118^) was determined using log rank tests; n ≥ 45 flies for each genotype. (D) Persistence of *L.mono* in *w*^1118^, *pickle*^ey/Df1^, and *spz*^rm7^ flies, measured at the indicated time points. All flies were injected with an identical initial dose of *L.mono* (∼1,500 CFU). Statistical significance was determined using a Mann-Whitney U test. (E and F) Kaplan-Meier plot showing the survival of female flies injected with (E) ∼200 CFU or (F) ∼10,000 CFU *P.ret*. Statistical significance between the survival of infected flies and a control *w*^1118^ strain was determined using log rank tests; n ≥ 45 flies for each genotype. ^∗^p ≤ 0.05, ^∗∗^p ≤ 0.01, ^∗∗∗^p ≤ 0.001, and ^∗∗∗∗^p ≤ 0.0001. See also Figure S6.

To study the physiological relevance of Pickle in selectively inhibiting RelN homodimers, we examined the response of *pickle* mutants to infection with the pathogenic bacteria *L. monocytogenes* (*L.mono*) and *P. rettgeri* (*P.ret*). Six hours after infection, both these bacteria activated also the Toll pathway in addition to the Imd pathway (Figures 7A, 7B, S6A, and S6B) (Buchon et al., 2009b, Gordon et al., 2005). Although these bacteria activated both the Imd and Toll pathways, some *AMPs* (*Defensin*) displayed different pathway dependency depending on the infecting bacteria. Induction of *AttD* in response to *L.mono* and *P.ret* infection was dependent solely on the Imd pathway (Figures 7A and 7B). *Defensin*, on the other hand, was solely Imd-dependent upon *L.mono* infection, whereas it was co-dependent on the Imd and Toll pathways following infection with *P.ret*. Interestingly, loss of *pickle* hyper-activated *AttD* and *Defensin* only when these *AMPs* were driven solely by RelN, such as following infection with *L.mono* (*AttD* and *Defensin*) and *P.ret* (*AttD*). Likewise, *c564::Gal4*-driven re-expression of *pickle* rescued the levels of *AttD* and *Defensin* expression to WT levels only when these *AMPs* were exclusively driven by RelN (Figure 7A). In contrast, loss of *pirk* caused hyper-activation *of AttD* and *Defensin* irrespective of the infecting bacteria, and irrespective of whether these *AMPs* were driven in an Imd- or Imd/Toll-dependent manner. Unlike *AttD* and *Defensin*, expression of *DiptA* and *DiptB* was insensitive to modulation by negative regulators such as *pirk* or *pickle*, quite possibly because these *AMPs* are already maximally induced. Our data are consistent with the notion that Pickle affects NF-κB target gene expression only when such genes are driven exclusively by RelN-only.

Next, we tested the ability of *pickle* to modulate the survival of flies infected with *L.mono*, *P.ret*, and *B. subtilis* (*B.sub*). *B.sub* is another pathogenic bacteria that activates both Imd and Toll pathways (Buchon et al., 2009b). Interestingly, *pickle*^ey/Df1^ mutant flies were significantly less susceptible to systemic infection with *L.mono*, *P.ret*, and *B.sub* (Figures 7C, 7E, 7F, and S6C). In some instances, *pickle* appeared haploinsufficient, as *pickle*^ey/+^ flies were significantly protected against *L.mono* and *P.ret* (∼200 colony-forming units [CFU]) infection compared with WT animals (*w*^1118^). Notably, this was dependent on bacterial dose, as at a higher dose (∼10,000 CFU), *pickle*^ey/+^ and WT flies rapidly succumbed to *P.ret* infection, whereas *pickle*^ey/Df1^ flies were significantly protected (Figure 7F). *c564::Gal4*-mediated re-expression of *pickle* in the fat body re-sensitized heterozygous flies to systemic bacterial infection (Figures 7C, 7E, and S6C), corroborating the specificity of the observed phenotype. The enhanced resistance of *pickle*^ey/Df1^ flies to *L.mono* was accompanied with a reduced bacterial load. Accordingly, *pickle*^ey/Df1^ flies harbored significantly fewer *L.mono* CFUs at 24 and 48 hr post-infection compared with WT controls (Figure 7D). Because *rel*^e20^ or *imd*^1^ mutant flies are acutely sensitive to infection with *B.sub*, *L.mono*, or *P.ret* (Figures S6D–S6F) (Buchon et al., 2009b, Mansfield et al., 2003), our data are consistent with a model whereby loss of *pickle* results in enhanced RelN-dependent immunity.

## Discussion

Tight regulation of NF-κB signaling is critical, as misbalanced and prolonged responses are detrimental to the host (Pasparakis, 2012). Here, we demonstrate that Pickle is required to prevent hyper-activation of Relish-dependent target genes. While loss of *pickle* improves host resistance to a variety of pathogenic bacteria, chronic inactivation of *pickle* compromises immune tolerance and shortens overall lifespan.

Pickle is a member of the IκB superfamily of proteins that selectively suppresses the production of Relish-dependent target genes. Like other IκB proteins, Pickle harbors C-terminal ARRs through which it binds to the RHD of Relish and inactivates Relish-mediated target gene expression, possibly via the recruitment of the histone deacetylase dHDAC1. Even though Pickle can bind to tethered DifˆRelN and dlˆRelN heterodimers, it suppresses NF-κB target gene expression only when such genes are driven solely by RelN. Accordingly, expression of Pickle strongly suppresses the transactivation ability of RelN as well as RelNˆRelN homodimers (Figures 6). By contrast, Pickle fails to inhibit Dif, dl, and linked dlˆRelN or DifˆRelN dimer combinations. Moreover, under conditions in which the Toll and Imd pathways are simultaneously activated, *pickle* exclusively influences induction of *AMPs* that are driven by RelN-only (Figures 6 and 7). Pickle, therefore, likely “skews” the output of both pathways via selective inhibition of genes solely transactivated by Relish. This is unlike Pirk, which regulates pathway flux, and does not selectively inhibit a specific subset of the NF-κB dimer repertoire.

Although homo- and heterodimers mediate diverse effects in mammalian systems (Hayden and Ghosh, 2008), it has been suggested that in *Drosophila* NF-κB proteins might mediate their effects as cooperating homodimers bound to distinct κB sites, rather than as heterodimers bound to a single site (Busse et al., 2007). Despite good evidence to suggest that heterodimers function in *Drosophila* (Han and Ip, 1999, Senger et al., 2004, Tanji et al., 2010), we cannot rule out the possibility that synergistic induction of *AMPs* is mediated by cooperating homodimers. Regardless of whether the *Drosophila* NF-κB proteins can act as either self-contained heterodimers or cooperating homodimers, our data demonstrate that Pickle inhibits *AMP* induction only when RelN is the only NF-κB member driving target gene expression. Under conditions in which *AMPs* are driven cooperatively by Dif and RelN, or Dif and dl, *AMP* production is insensitive to the presence of Pickle.

Pickle’s ability to bias the output of certain Relish-dependent target genes, namely, those that are driven solely by RelN:RelN, has important physiological consequences. In the short term, loss of *pickle* enhances expression of RelN target genes, significantly boosting the host defense from infection with pathogenic bacteria. Although we observed elevated levels of several *AMPs* in *pickle* mutant flies, mere hyper-activation of these *AMPs* was not the only reason these animals were protected. *pirk* mutant animals similarly hyper-activated these *AMP* genes, yet these animals were unable to fend off *L.mono*, *P.ret*, and *B.sub*. The difference between loss of *pickle* and loss of *pirk* is likely due to the differential regulation of Imd signaling. Because Pirk regulates Imd signaling at the level of the receptor (or Imd) (Aggarwal et al., 2008, Kleino et al., 2008, Lhocine et al., 2008), Pirk is unable to skew the Imd and Toll signaling outputs toward a subset of NF-κB target genes that are driven by a particular NF-κB dimer combination. Although in the short term, loss of *pickle* appears to be beneficial for immune defense against certain pathogenic bacteria (*L.mono*, *P.ret*, and *B.sub*), in the long run, chronic inactivation of pickle results in loss of immune tolerance and shortened lifespan. Pickle, therefore, allows for a balanced immune response that protects from pathogenic microbes while permitting the establishment of beneficial commensal host-microbe relationships. At present little is known how the host tolerates commensal bacteria while mounting a full response to others. Our observations are consistent with a model in which Pickle acts as an immune modulator that balances the complex relationship between host resistance to pathogens and immune tolerance to microbiota. Because breakdown of this balance contributes to the development of immune-related pathologies (Pasparakis, 2009), further dissection of Pickle’s unique regulatory action may aid our understanding of how aberrant NF-κB activity contributes to dysfunction of the immune system.

## Experimental Procedures

### Fly Stocks, Husbandry, and Bacterial Cultures

Flies were kept at 25°C, unless stated otherwise. A full list of all genotypes used for each figure can be found in Table S2. Bacterial cultures were initiated from single colonies grown on LB plates. Small volumes of the starter cultures were then diluted at least 1:1,000 (so as to have an near undetectable optical density [OD]) and cultured up to the desired OD on the day of the experiment. For hk *M.lut* and *E.coli* solutions, bacteria were suspended in sterile PBS and subsequently hk for 10 min at 95°C in a heating block. Heat-killed bacterial solutions were diluted so as to enable the injection of approximately equal numbers of *E.coli* (hk) and *M.lut* (hk). Preparations were then aliquoted and frozen at −80°C for repeat use of identical hk bacterial preparations. See Supplemental Experimental Procedures for details.

### Systemic Infection Experiments and Survival

Three- to eight-day-old adult flies were used for infection experiments. Systemic infection was performed by injecting flies with 13.8 nl of a cultured bacterial solution, PBS, or hk bacteria resuspended in PBS, using the Nanoject II (Drummond Scientific). Flies were then incubated at 25°C, transferred to fresh vials every day, and collected and examined at different time points for qRT-PCR, CFU counts, and survival analysis.

### Oral Infection and Bleomycin Treatments and Generation of Axenic Flies

Oral infections and treatments were performed as previously reported (Buchon et al., 2009a), with some modifications. Briefly, 5- to 7-day-old female flies were raised, starved, and fed on a Whatman filter paper covered by 150 μl of an infection solution (*Ecc15* at OD 100 or *P.e* at OD 50) or 250 μg/ml bleomycin solution (Sigma) containing 2.5% sucrose. See Supplemental Experimental Procedures for details.

### Generation of Axenic Flies

Freshly laid eggs (≤5 hr old) were collected from grape juice agar plates. Embryos were rinsed in 1× PBS, and any hatched larvae or loose agar pieces were removed with sterile forceps. All subsequent steps were performed in a sterilized laminar flow hood. Embryos were surface-sterilized by 70% ethanol and then by 5% sodium hypochlorite for 10 min, followed by three washes with sterile water, and then aseptically transferred to sterile food in a small amount of 100% ethanol. Adult female flies (about 7 days old) were collected for midgut dissection.

### Lifespan Analysis

Five virgins *5966::GS* homozygotes were crossed to one male with the indicated genotypes. Ten crosses were set up per genotype. Progenies were collected and allowed to mate for 2 days. Male siblings were then separated (20 flies per vial). Flies were treated with RU486, as previously described (Guo et al., 2014), with some modifications. See Supplemental Experimental Procedures for details.

### Bacterial Load

The bacterial load was established as previously described (Khalil et al., 2015). Fly homogenates were serially diluted (10-fold), and CFUs were counted manually. Ten flies were analyzed per genotype and experimental repeat. A “mock” procedure lacking injected bacteria was performed in each experiment repeat. No CFUs were detectable following this “mock” procedure.

### qRT-PCR and Primer Sequences

qRT-PCR was performed as previously described (Meinander et al., 2012), with some modifications. For whole-fly analysis in Figures 3, 4, and S3, pools of 15 male and 15 female flies per sample were analyzed. For whole-fly analysis in Figures 6, 7, S5, and S6, pools of 5 female flies per sample were analyzed. For midgut analysis, pools of 15–20 dissected female midguts were analyzed. The amount of mRNA detected was normalized to control *rp49* mRNA values. In Figures 5, 6, S4, and S5, the ΔCt^sample^ /ΔCt^rp49^ ratios are indicated to allow comparison of the actual expression levels. For the remaining figures, relative ΔCt^sample^/ΔCt^rp49^ ratios of WT controls were set at 100%, and the fold differences were calculated using the ΔΔCt method. See Supplemental Experimental Procedures for additional details and primer sequences.

### Tissue Culture and Treatments

*Drosophila* S2^∗^ cells were a kind gift from Neal Silverman. S2^∗^ cells were cultured at 23°C in Schneider’s *Drosophila* medium (Gibco), supplemented with 10% fetal bovine serum, 60 μg/ml penicillin, and 100 μg/ml streptomycin. RNAi knockdown was performed as described previously (http://www.flyrnai.org/DRSC-PRR.html). Transfections were performed using Effectine (Qiagen) or calcium phosphate protocol (Clontech) according to the manufacturer’s instructions. See Supplemental Experimental Procedures for details.

### Immunoprecipitation, Nuclear and Cytoplasmic Fractionation, and Western Blot Analysis

Immunoprecipitation and western blot analysis were performed as previously described (Meinander et al., 2012), with some modifications. Cytoplasmic and nuclear fractions were separated via combined used of centrifugation and cytoplasmic and nuclear extraction buffers. See Supplemental Experimental Procedures for details.

### Sequence Collection, Phylogenetic Analysis, and Model Constructions

Analysis was performed as previously described (Basith et al., 2013). The 3D model of Pickle was built using ANK-N5C (Protein Data Bank [PDB]: 4O60) as template, which shares a sequence identity of 26.8%. See Supplemental Experimental Procedures for details.

## Author Contributions

O.M. designed and performed the experiments. X.L. and N.B. conducted and supervised the experiments involving *Drosophila* midgut. C.D. provided advice throughout systemic infection experiments. C.R. performed preliminary experiments investigating the immune function of *pickle*. T.T. gave advice throughout western blotting experiments. A.C. and G.P. performed preliminary experiments investigating the pathological impact of *pickle*. H.C. performed preliminary experiments involving *Drosophila* midgut. S.B. and S.C. performed bioinformatics analysis. O.M. and P.M. designed the study and wrote the manuscript.

## Supplemental Information

Document S1. Supplemental Experimental Procedures and Figures S1–S6 Document S1. Supplemental Experimental Procedures and Figures S1–S6

Table S1. Taxa Terminologies of Vertebrate and Invertebrate IκB Proteins Used in This Study, Related to Figure 2 Table shows name of species, species abbreviation and respective IκB gene sequence identifiers. Table S1. Taxa Terminologies of Vertebrate and Invertebrate IκB Proteins Used in This Study, Related to Figure 2

Table S2. Fly Genotypes Used in This Study, Related to Experimental Procedures Table S2. Fly Genotypes Used in This Study, Related to Experimental Procedures

Document S2. Article plus Supplemental Information Document S2. Article plus Supplemental Information
