# Supplementary material for: Signal Integration by the IκB Protein Pickle Shapes Drosophila Innate Host Defense
Source: Cell Host Microbe. 2016 Sep 14;20(3):283–95. doi: 10.1016/j.chom.2016.08.003 (PMC5026699; doi:10.1016/j.chom.2016.08.003)
Supplement: Document S1. Supplemental Experimental Procedures and Figures S1–S6 [file mmc1.pdf]

Cell Host & Microbe, Volume *20*

Supplemental Information

Signal Integration by the IκB Protein Pickle Shapes *Drosophila* Innate Host Defense

Otto Morris, Xi Liu, Celia Domingues, Christopher Runchel, Andrea Chai, Shaherin Basith, Tencho Tenev, Haiyang Chen, Sangdun Choi, Giuseppa Pennetta, Nicolas Buchon, and Pascal Meier

Supplementary Figures and Legends

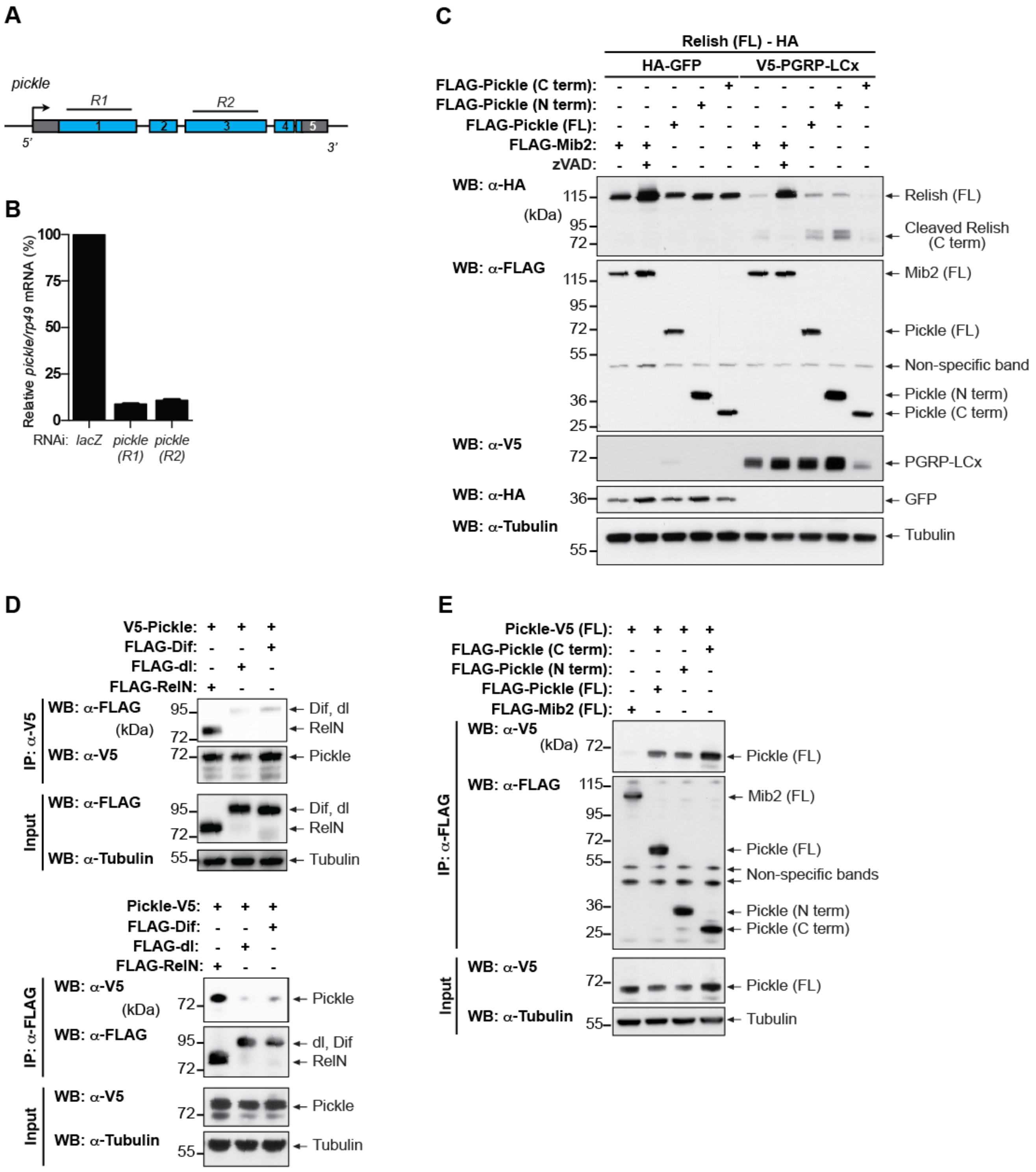

Figure S1, related to Figure 1. Pickle negatively regulates Relish

(A) Schematic representation depicting the regions that are being targeted by *pickle*'s *R1* and *R2* dsRNAs. **(B)** RT-qPCR analysis of relative *pickle* mRNA levels from S2* cells incubated with the indicated dsRNAs. **(C)** Co-expression of Pickle does not alter Relish cleavage following activation of Imd signalling by PGRP-LCx. The indicated proteins were co-expressed in S2* cells and Relish cleavage assessed via western blot. Treatment with the caspase inhibitor zVAD-FMK served as control. **(D)** Pickle selectively binds to RelN and does not interact with dl and Dif. The indicated FLAG- and V5-tagged proteins were co-expressed in S2* cells. Reciprocal FLAG and V5 immuno-precipitations were performed and binding assessed via western blot. **(E)** Pickle homo-oligomerizes. V5-tagged Pickle (FL) was expressed in S2* cells along with the indicated FLAG-tagged proteins. FLAG immuno-precipitation was performed, and binding assessed via western blot.

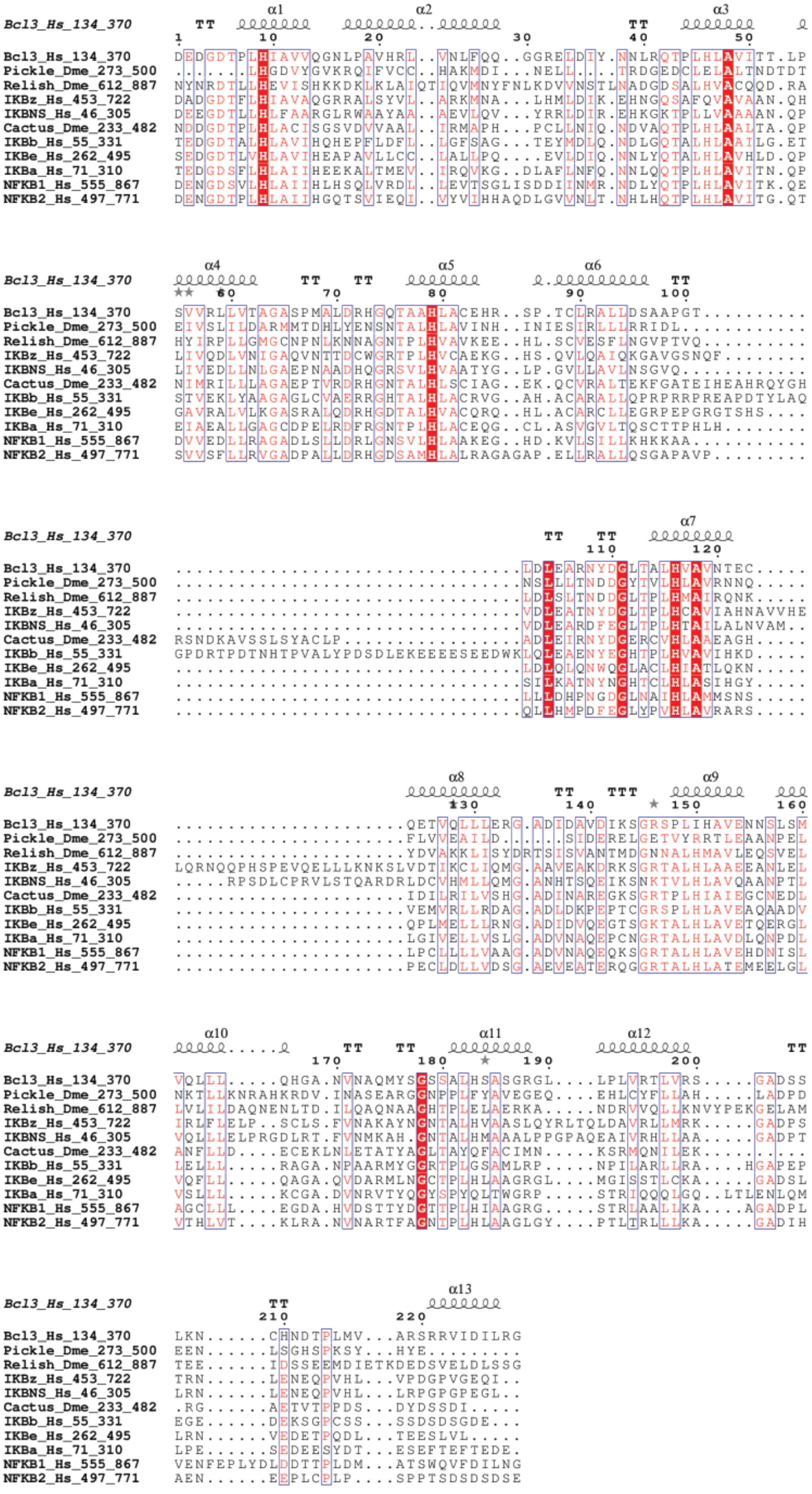
Bcl3_Hs_134_370
α1 α2 α3
1 10 20 30 40 50
Bcl3_Hs_134_370 DEDGDTPLHIAVVQGNLPAVHRL..VNLFQQ..GGRELDIY.NNLRQTPLHLAVITT.LP
Pickle_Dme_273_500 ......LHGDVYGVKRQIFVCC..HAKMDI...NELL....TRDGEDCLELALTNDTDT
Relish_Dme_612_887 NYNRDTLLHEVISHKKDKLKLAIQTIQVMNYFNLKDVVNSTLNADGDSALHVACQQD.RA
IKBz_Hs_453_722 DADGDTFLHIAVAQGRRALSYVL..ARKMNA...LHMLDIK.EHNGQSAFQVAVAAN.QH
IKBNS_Hs_46_305 DEEGDTLLHLFAARGLRWAAYAA..AEVLQV...YRRLDIR.EHKGKTPLLVAAAAN.QP
Cactus_Dme_233_482 NDDGDTPLHLACISGSVDVVAAL..IRMAPH...PCLLNIQ.NDVAQTPLHLAALTA.QP
IKBb_Hs_55_331 TEDGDTALHLAVIHQHEPFLDFL..LGFSAG...TEYMDLQ.NDLGQTALHLAAILG.ET
IKBe_Hs_262_495 SEDGDTLVHLAVIHEAPAVLLCC..LALLPQ...EVLDIQ.NNLYQTALHLAVHLD.QP
IKBa_Hs_71_310 TEDGDSFLHLAIIHEEKALTMEV..IRQVKG..DLAFLNFQ.NNLQQTPLHLAVITN.QP
NFKB1_Hs_555_867 DENGDSVLHLAIIHLHSQLVRDL..LEVTSGLISDDIINMR.NDLYQTPLHLAVITK.QE
NFKB2_Hs_497_771 DENGDTPLHLAIIHGQTSVIEQI..VYVIHHAQDLGVVNLT.NHLHQTPLHLAVITG.QT
Bcl3_Hs_134_370
α4 α5 α6
60 70 80 90 100
Bcl3_Hs_134_370 SVVRLLVTAGASPMALDRHGQTAAHLACEHR..SP.TCLRALLDSAAPGT..........
Pickle_Dme_273_500 EIVSLILDARMMTDHLYENSNTALHLAVINH..INIESIRLLLRRIDL...........
Relish_Dme_612_887 HYIRPLLGMGCNPNLKNNAGNTPLHVAVKEE..HL.SCVESFLNGVPTVQ..........
IKBz_Hs_453_722 LIVQDLVNIGAQVNTTDCWGRTPLHVCAEKG..HS.QVLQAIQKGAVGSNQF........
IKBNS_Hs_46_305 LIVEDLLNLGAEPNAADHQGRSVLHVAATYG..LP.GVLLAVLNSGVQ............
Cactus_Dme_233_482 NIMRILLLAGAEPTVRDRHGNTALHLSCIAG..EK.QCVRALTEKFGATEIHEAHRQYGH
IKBb_Hs_55_331 STVEKLYAAGAGLCVAERRGHTALHLACRVG..AH.ACARALLQPRPRRPREAPDTYLAQ
IKBe_Hs_262_495 GAVRALVLKGASRALQDRHGDTALHVACQRQ..HL.ACARCLLEGRPEPGRGTSHS....
IKBa_Hs_71_310 EIAEALLGAGCDPELRDFRGNTPLHLACEQG..CL.ASVGVLTQSCTTPHLH........
NFKB1_Hs_555_867 DVVEDLLRAGADLSLLDRLGNSVLHLAAKEG..HD.KVLSILLKHKKAA...........
NFKB2_Hs_497_771 SVVSFLLRVGADPALLDRHGDSAMHLALRAGAGAP.ELLRALLQSGAPAVP.........
Bcl3_Hs_134_370
α7
110 120
Bcl3_Hs_134_370 .................................LDLEARNYDGLTALHVAVNTEC.....
Pickle_Dme_273_500 .................................NSLLLTNDDGYTVLHLAVRNNQ.....
Relish_Dme_612_887 .................................LDLSLTNDDGLTPLHMAIRQNK.....
IKBz_Hs_453_722 .................................VDLEATNYDGLTPLHCAVIAHNAVVHE
IKBNS_Hs_46_305 .................................VDLEARDFEGLTPLHTAILALNVAM..
Cactus_Dme_233_482 RSNDKAVSSLSYACLP.................ADLEIRNYDGERCVHLAAEAGH.....
IKBb_Hs_55_331 GPDRTPDTNHTPVALYPDSDLEKEEEESEEDWKLQLEAENYEGHTPLHVAVIHKD.....
IKBe_Hs_262_495 .................................LDLQLQNWQGLACLHIATLQKN.....
IKBa_Hs_71_310 .................................SILKATNYNGHTCLHLASIHGY.....
NFKB1_Hs_555_867 .................................LLLDHPNGDGLNAIHLAMMSNS.....
NFKB2_Hs_497_771 .................................QLLHMPDFEGLYPVHLAVRARS.....
Bcl3_Hs_134_370
α8 α9
130 140 150 160
Bcl3_Hs_134_370 ....QETVQLLLERG.ADIDAVDIKSGRSPLIHAVENNSLSM
Pickle_Dme_273_500 ....FLVVEAILD.....SIDERELGETVYRRTLEAANPEL
Relish_Dme_612_887 ......................YDVAKKLISYDRTSISVANTMDGNNALHMAVLEQSVEL
IKBz_Hs_453_722 LQRNQQPHSPEVQELLLKNKSLVDTIKCLIQMG.AAVEAKDRKSGRTALHLAAEEANLEL
IKBNS_Hs_46_305 .....RPSDLCPRVLSTQARDRLDCVHMLLQMG.ANHTSQEIKSNKTVLHLAVQAANPTL
Cactus_Dme_233_482 ....IDILRILVSHG.ADINAREGKSGRTPLHIAIEGCNEDL
IKBb_Hs_55_331 ....VEMVRLLRDAG.ADLDKPEPTCGRSPLHLAVEAQAADV
IKBe_Hs_262_495 ....QPLMELLLRNG.ADIDVQEGTSGKTALHLAVETQERGL
IKBa_Hs_71_310 ....LGIVELLVSLG.ADVNAQEPCNGRTALHLAVDLQNPDL
NFKB1_Hs_555_867 ....LPCLLLLVAAG.ADVNAQEQKSGRTALHLAVEHDNISL
NFKB2_Hs_497_771 ....PECLDLLVDSG.AEVEATERQGGRTALHLATEMEELGL
Bcl3_Hs_134_370
α10 α11 α12
170 180 190 200
Bcl3_Hs_134_370 VQLLL.....QHGA.NVNAQMYSGSSALHSASGRGL....LPLVRTLVRS.....GADSS
Pickle_Dme_273_500 NKTLLKNRAHKRDV.INASEARGGNPPLFYAVEGEQ....EHLCYFLLAH.....LADPD
Relish_Dme_612_887 LVLILDAQNENLTD.ILQAQNAAGHTPLELAERKA.....NDRVVQLLKNVYPEKGELAM
IKBz_Hs_453_722 IRLFLELP..SCLS.FVNAKAYNGNTALHVAASLQYRLTQLDAVRLLMRK.....GADPS
IKBNS_Hs_46_305 VQLLLELPRGDLRT.FVNMKAH.GNTALHMAAALPPGPAQEAIVRHLLAA.....GADPT
Cactus_Dme_233_482 ANFLLD....ECEKLNLETATYAGLTAYQFACIMN.....KSRMQNILEK..........
IKBb_Hs_55_331 LELLL.....RAGA.NPAARMYGGRTPLGSAMLRP.....NPILARLLRA.....HGAPEP
IKBe_Hs_262_495 VQFLL.....QAGA.QVDARMLNGCTPLHLAAGRGL....MGISSTLCKA.....GADSL
IKBa_Hs_71_310 VSLLL.....KCGA.DVNRVTYQGYSPYQLTWGRP.....STRIQQQLGQ..LTLENLQM
NFKB1_Hs_555_867 AGCLLL....EGDA.HVDSTTYDGTTPLHIAAGRG.....STRLAALLKA....AGADPL
NFKB2_Hs_497_771 VTHLVT....KLRA.NVNARTFAGNTPLHLAAGLGY....PTLTRLLLKA.....GADIH
Bcl3_Hs_134_370
α13
210 220
Bcl3_Hs_134_370 LKN......CHNDTPLMV...ARSRRVIDILRG
Pickle_Dme_273_500 EEN......LSGHSPKSY...HYE........
Relish_Dme_612_887 TEE......IDSSEEMDIETKDEDSVELDLSSG
IKBz_Hs_453_722 TRN......LENEQPVHL...VPDGPVGEQI..
IKBNS_Hs_46_305 LRN......LENEQPVHL...LRPGPGPEGL..
Cactus_Dme_233_482 .RG......AETVTPPDS...DYDSSDI.....
IKBb_Hs_55_331 EGE......DEKSGPCSS...SSDSDSGDE...
IKBe_Hs_262_495 LRN......VEDETPQDL...TEESLVL.....
IKBa_Hs_71_310 LPE......SEDEESYDT...ESEFTEFTEDE.
NFKB1_Hs_555_867 VENFEPLYDLDDTTPLDM...ATSWQVFDILNG
NFKB2_Hs_497_771 AEN......EEPLCPLP....SPPTSDSDSDSE

Figure S2, related to Figure 2. Phylogenetic relationship of Pickle with other IκB family members

Sequence comparisons of IκB ankyrin repeat regions (ARRs). The multiple sequence alignment of representative IκB members is shown. The amino acid numbers shown correspond to the ARR of the indicated IκB proteins. The highly conserved regions in the sequence alignment of IκB ARRs are represented in red blocks. The secondary structure prediction in relation to the Bcl-3 structure is shown at the top of the sequence alignment.

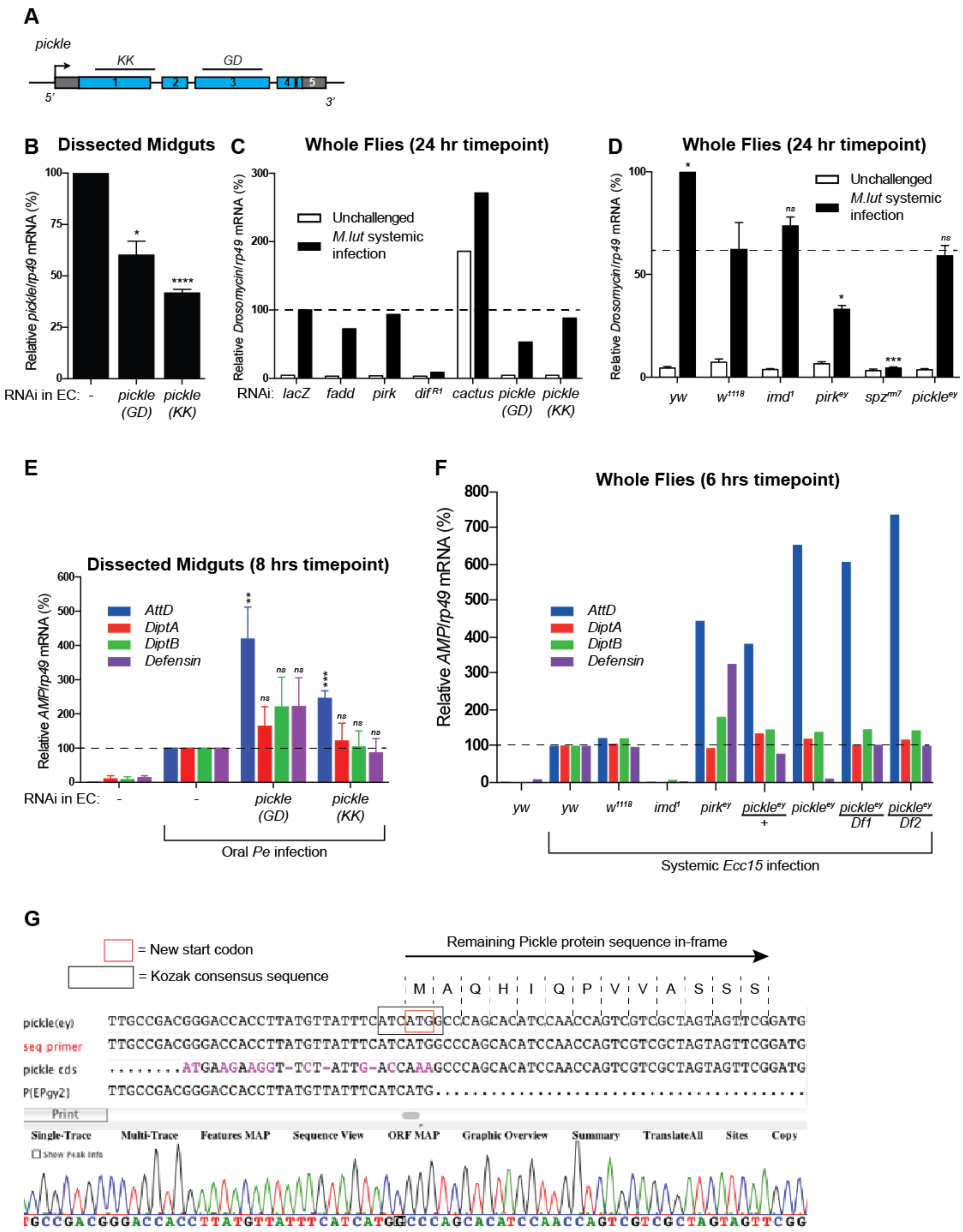
A
pickle
KK
GD
1
2
3
4
5
5'
3'
B
Dissected Midguts
Relative pickle/rp49 mRNA (%)
100
75
50
25
0
*
****
RNAi in EC: - pickle (GD) pickle (KK)
C
Whole Flies (24 hr timepoint)
Relative Drosomycin/rp49 mRNA (%)
300
200
100
0
Unchallenged
M.lut systemic infection
RNAi: lacZ fadd pirk dif R1 cactus pickle (GD) pickle (KK)
D
Whole Flies (24 hr timepoint)
Relative Drosomycin/rp49 mRNA (%)
100
50
0
Unchallenged
M.lut systemic infection
*
ns
ns
*
***
ns
yw w1118 imd1 pirkey spzrm7 pickleey
E
Dissected Midguts (8 hrs timepoint)
Relative AMP/rp49 mRNA (%)
600
500
400
300
200
100
0
AttD
DiptA
DiptB
Defensin
**
ns
ns
ns
***
ns
ns
ns
RNAi in EC: - - pickle (GD) pickle (KK)
Oral Pe infection
F
Whole Flies (6 hrs timepoint)
Relative AMP/rp49 mRNA (%)
800
700
600
500
400
300
200
100
0
AttD
DiptA
DiptB
Defensin
yw yw w1118 imd1 pirkey pickleey/+ pickleey pickleey/Df1 pickleey/Df2
Systemic Ecc15 infection
G
= New start codon
= Kozak consensus sequence
Remaining Pickle protein sequence in-frame
M A Q H I Q P V V A S S S
pickle(ey) TTGCCGACGGGACCACCTTATGTTATTTCATCATGGCCCAGCACATCCAACCAGTCGTCGCTAGTAGTTCGGATG
seq primer TTGCCGACGGGACCACCTTATGTTATTTCATCATGGCCCAGCACATCCAACCAGTCGTCGCTAGTAGTTCGGATG
pickle cds ....ATGAAGAAGGT-TCT-ATTG-ACCAAAGCCCAGCACATCCAACCAGTCGTCGCTAGTAGTTCGGATG
P{EPgy2} TTGCCGACGGGACCACCTTATGTTATTTCATCATG..
Print
Single-Trace Multi-Trace Features MAP Sequence View ORF MAP Graphic Overview Summary TranslateAll Sites Copy
Show Peak Info
TGCCGACGGGACCACCTTATGTTATTTCATCATGGCCCAGCACATCCAACCAGTCGTCGCTAGTAGTTCGG

Figure S3, related to Figure 3. Loss of *pickle* causes hyper-induction of *AMPs* following infection with Gram-negative bacteria

(A) Scheme representation depicting the dsRNA regions that are targeted in the *pickle* RNAi lines *(GD)* and *(KK)*. **(B-F)** RT-qPCR analysis of the indicated flies. (B) Relative *pickle* mRNA levels from dissected midguts. RNAi knockdown was restricted to enterocytes (EC) using *myo::Gal4*. (C, D) Relative *Drosomycin* mRNA levels from the indicated flies before and after 24 hrs of infection with *M.lut* (~200 CFU). (C) RNAi of the indicated target genes was driven in the fat body (FB) using *c564::Gal4*. (E) *AMP* mRNA levels of dissected midguts before and after 8 hrs of oral infection with *P.e*. RNAi knockdown was restricted to enterocytes (EC) using *myo::Gal4*. (F) Relative *AMP* mRNA levels from whole flies before and after 6 hrs of infection with *Ecc15* (~2000 CFU). **(G)** Sequence analysis of the $pickle^{ey}$ allele depicting the position of the ATG at end of *P[EPgy2].* Note the ATG at the end of the UAS promoter is in-frame with the remaining *pickle* coding sequence. Results were expressed as % of a control sample (marked with dotted line). Unless otherwise indicated, P values are measured from control these via an unpaired, Student *t*-test. Histograms depict mean ± SEM of three (B, D, E) or two (C, F) biological repetitions. * $P \leq 0.05$, ** $P \leq 0.01$, *** $P \leq 0.001$ and **** $P \leq 0.0001$.

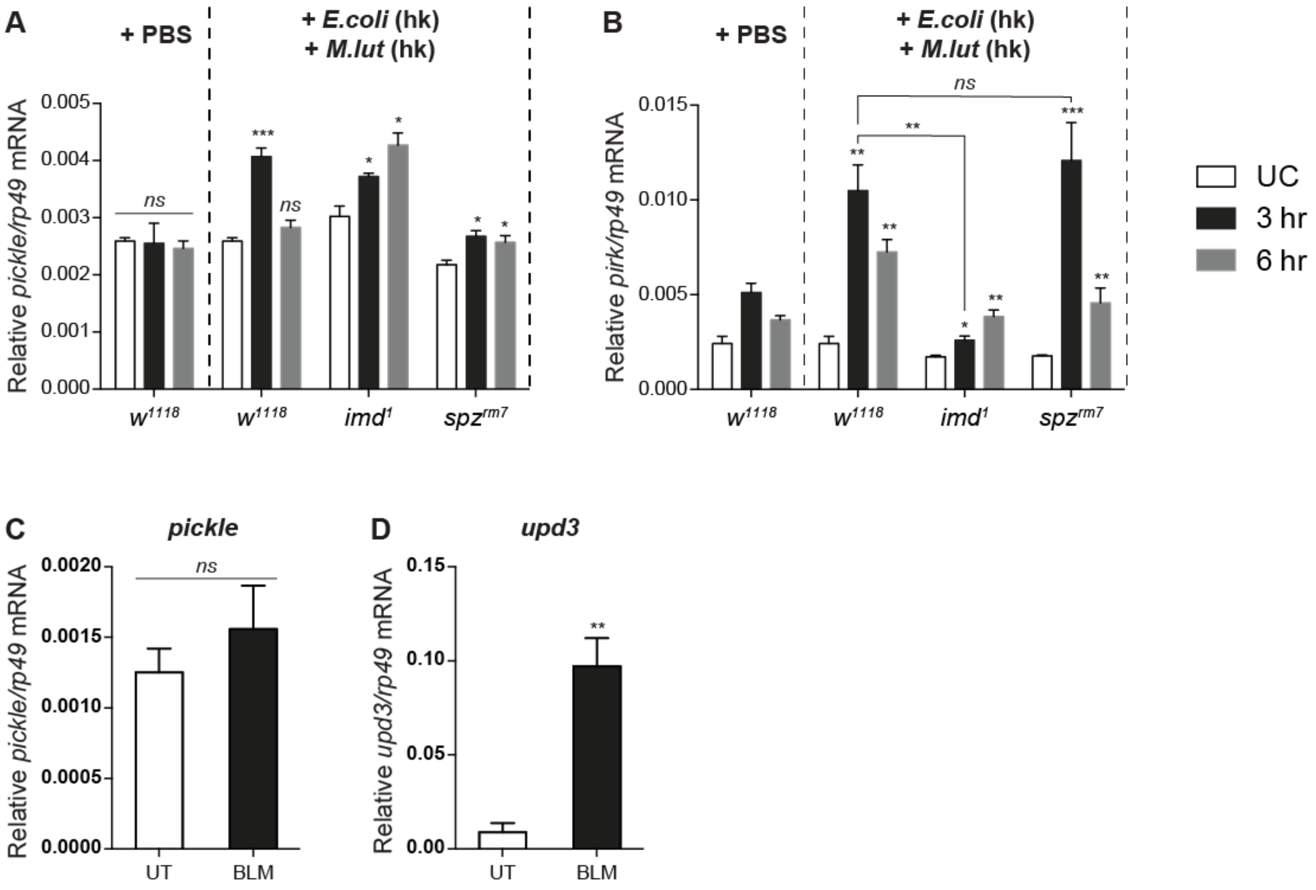

Figure S4, related to Figure 5. *pickle* expression is induced in response to commensal and infectious bacteria

(A-D) RT-qPCR analysis of *pickle, pirk* or *upd3* transcript levels. Relative (A) *pickle* and (B) *pirk* mRNA levels from whole flies unchallenged (UC) or injected with PBS or a heat-killed *E.coli* + *M.lut* mixture. **(C, D)** Unlike the stress-response cytokine *upd3*, *pickle* was not transcriptionally induced by damage. Relative (C) *pickle* and (D) *upd3* mRNA levels from dissected midguts of Canton S flies before and after (24 hrs) treatment with the tissue damaging agent bleomycin (BLM). Histograms depict mean ± SEM of three biological repeats, and statistical significance was calculated using an unpaired, student *t*-test. * $P \leq 0.05$, ** $P \leq 0.01$, *** $P \leq 0.001$ and **** $P \leq 0.0001$.

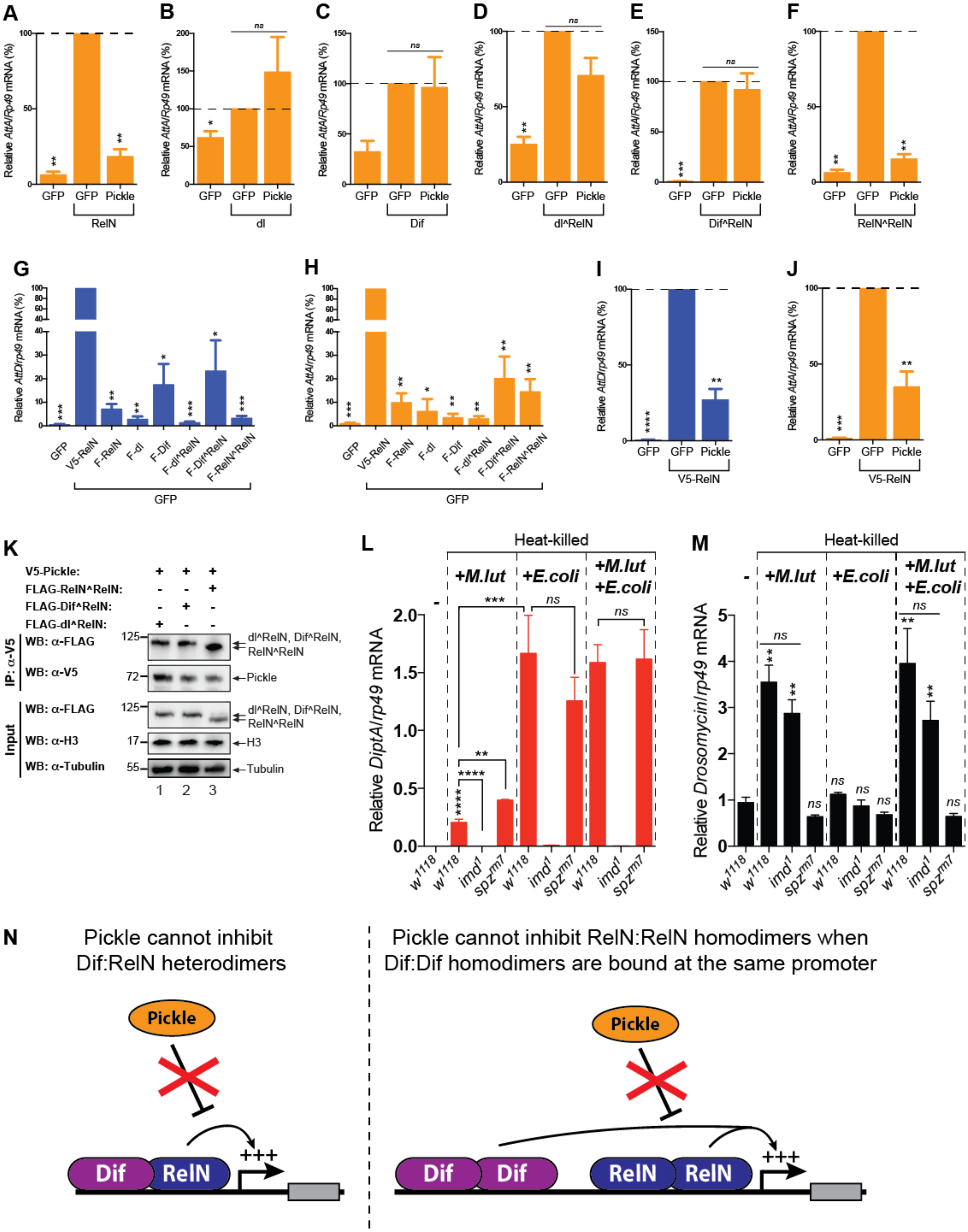
A
Relative AttA/Rp49 mRNA (%)
GFP GFP Pickle
RelN
B
ns
dl
C
Dif
D
dl^RelN
E
Dif^RelN
F
RelN^RelN
G
Relative AttD/rp49 mRNA (%)
GFP V5-RelN F-RelN F-dl F-Dif F-dl^RelN F-Dif^RelN F-RelN^RelN
GFP
H
Relative AttA/rp49 mRNA (%)
I
V5-RelN
J
K
V5-Pickle:
FLAG-RelN^RelN:
FLAG-Dif^RelN:
FLAG-dl^RelN:
IP: α-V5
WB: α-FLAG
WB: α-V5
Input
WB: α-FLAG
WB: α-H3
WB: α-Tubulin
dl^RelN, Dif^RelN, RelN^RelN
Pickle
H3
Tubulin
1 2 3
L
Heat-killed
+M.lut
+E.coli
+M.lut +E.coli
Relative DiptA/rp49 mRNA
w1118 w1118 imd1 spzrm7 w1118 imd1 spzrm7 w1118 imd1 spzrm7
M
Relative Drosomycin/rp49 mRNA
N
Pickle cannot inhibit Dif:RelN heterodimers
Pickle cannot inhibit RelN:RelN homodimers when Dif:Dif homodimers are bound at the same promoter
Pickle
Dif
RelN
+++

Figure S5, related to Figure 6. Pickle selectively inhibits RelN homodimers

(A-F) Relative *AttA* mRNA levels of S2* cells transiently transfected with the indicated constructs. All proteins are FLAG(F)-tagged at their N-termini. Histograms depict mean ± SEM of three biological repeats. Results are expressed as % of induced GFP control samples of each experiment, and statistical significance was measured from these via an unpaired, two-tailed Student *t*-test. **(G-J)** Relative *AttD* and *AttA* mRNA levels of S2* cells transiently transfected with the indicated constructs. Results are expressed as % of the V5-RelN + GFP sample in each experiment, and statistical significance was measured from this via an unpaired, Student *t*-test. **(K)** Pickle binds to dl^RelN, Dif^RelN and RelN^RelN. The indicated proteins were co-expressed in S2* cells. V5 immuno-precipitation was performed and binding was assessed via western blot. **(L, M)** Relative (L) *DiptA* or (M) *Drosomycin* levels from unchallenged flies or flies injected with heat-killed *E.coli* and/or heat-killed *M.lut* (6 hrs). Unless otherwise indicated, statistical significance is measured from unchallenged w^{1118} flies via an unpaired, Student *t*-test. **(N)** Depicted are two possible models that help to explain Pickle's ability to selectively inhibit RelN only when it acts alone, and not when it operates in concert with Dif. * $P \leq 0.05$, ** $P \leq 0.01$, *** $P \leq 0.001$ and **** $P \leq 0.0001$.

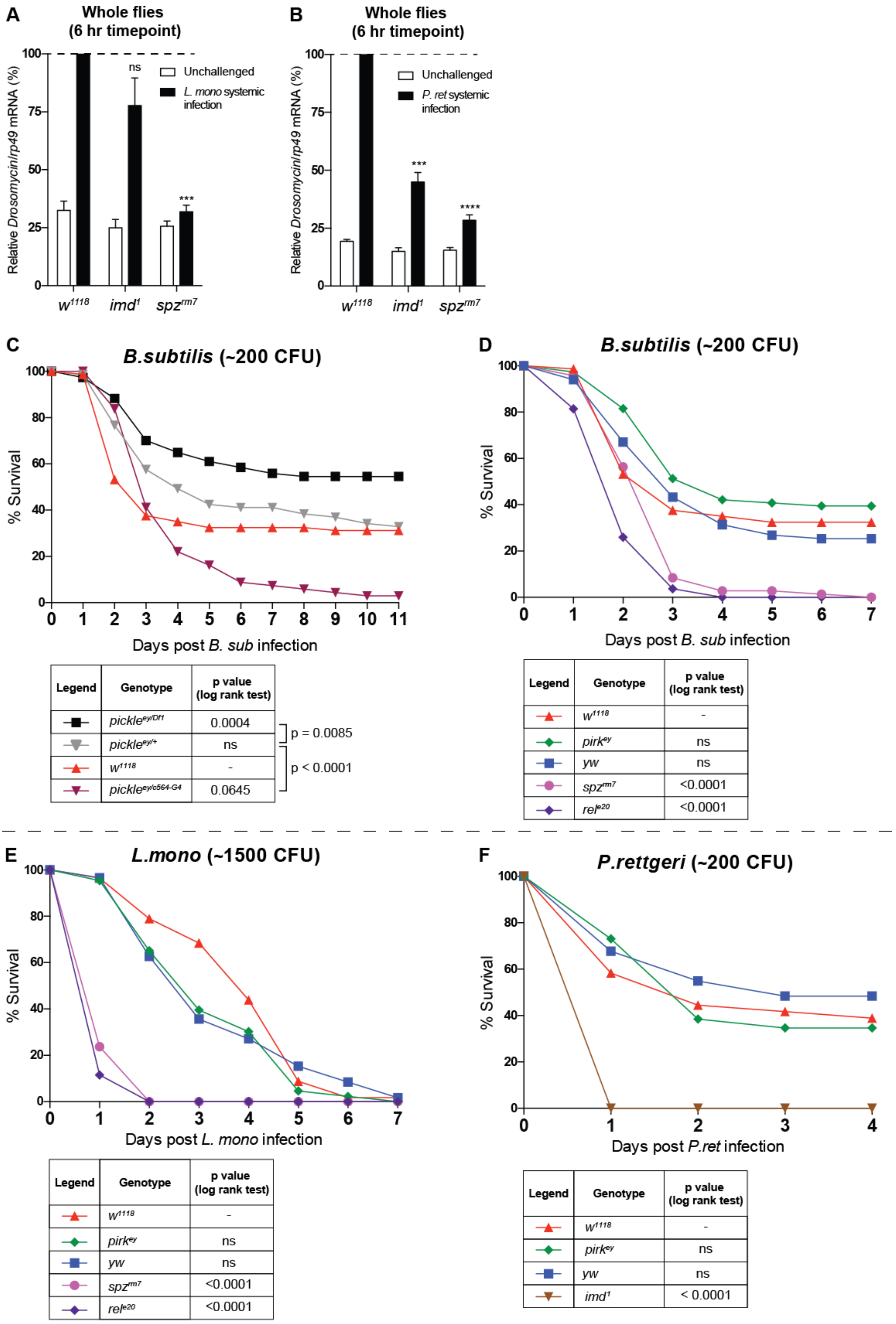
A
Whole flies
(6 hr timepoint)
Relative Drosomycin/rp49 mRNA (%)
100
75
50
25
0
ns
***
Unchallenged
L. mono systemic infection
w1118
imd1
spzrm7
B
Whole flies
(6 hr timepoint)
Relative Drosomycin/rp49 mRNA (%)
***
****
Unchallenged
P. ret systemic infection
w1118
imd1
spzrm7
C
B.subtilis (~200 CFU)
% Survival
Days post B. sub infection
Legend | Genotype | p value (log rank test)
pickleey/Df1 | 0.0004
pickleey/+ | ns
w1118 | -
pickleey/c564-G4 | 0.0645
p = 0.0085
p < 0.0001
D
B.subtilis (~200 CFU)
% Survival
Days post B. sub infection
Legend | Genotype | p value (log rank test)
w1118 | -
pirkey | ns
yw | ns
spzrm7 | <0.0001
rele20 | <0.0001
E
L.mono (~1500 CFU)
% Survival
Days post L. mono infection
Legend | Genotype | p value (log rank test)
w1118 | -
pirkey | ns
yw | ns
spzrm7 | <0.0001
rele20 | <0.0001
F
P.rettgeri (~200 CFU)
% Survival
Days post P.ret infection
Legend | Genotype | p value (log rank test)
w1118 | -
pirkey | ns
yw | ns
imd1 | < 0.0001

Figure S6, related to Figure 7. Loss of *pickle* improves host resistance to pathogenic bacteria

(A, B) RT-qPCR analysis of *Drosomycin* mRNAs of samples from the indicated flies, before and 6 hrs post-systemic infection with (A) *L. monocytogenes* (~1500 CFU) and (B) *P.rettgeri* (~10000 CFU). Histograms depict mean ± SEM of three biological repeats. Results are expressed as % of the induced levels of control flies (w^{1118}) in each experiment (marked with a dotted line), and statistical significance was measured from these via unpaired, two-tailed Student's *t*-test. **(C-F)** Kaplan-Meier plot showing the survival of female flies injected with (C, D) *B. subtilis* (~200 CFU), (E) *L. monocytogenes* (~1500 CFU) and (F) *P.rettgeri* (~200 CFU). Statistical significance between infected flies and a control w^{1118} strain were determined by log-rank tests. n ≥ 45 flies for each genotype except rel^{e20} and imd^{1} genotypes, for which n ≥ 25 flies. * $P \leq 0.05$, ** $P \leq 0.01$, *** $P \leq 0.001$ and **** $P \leq 0.0001$.

Supplementary Table S1, related to Figure 2. Table listing taxa terminologies of vertebrate and invertebrate IκB proteins used in this study

Table shows name of species, species abbreviation and respective IκB gene sequence identifiers.

Supplementary Table S2, related to Experimental Procedures. Table listing fly genotypes used in this study

Supplemental Experimental Procedures

Fly stocks, husbandry and bacterial cultures

Canton S, w^{1118} and *yw* flies were used as control flies as appropriate. *UAS-lacZ* flies were a kind gift from M. Miura (University of Tokyo). *UAS-pirk-IR* and *pirk^{ey}* flies were described previously (Lhocine et al., 2008). *UAS-fadd-IR* (VDRC 100333), *UAS-pickle(GD)-IR* (VDRC 34936) and *UAS-pickle(KK)-IR* flies (VDRC 106292) were obtained from VDRC stock centre. Note that VDRC 34936 is no longer publically available at VDRC. *Df(2L)Exel7006, Df(2L)BSC481, pickle^{ey}*, *UAS-relish-IR (TRIP 33661), UAS-cactus-IR (TRIP 34775), UAS-dif(R1)-IR (TRIP 30513), UAS-dif(R2)-IR (TRIP 29514), UAS-dl(R1)-IR (TRIP 27650)* and *UAS-dl(R2)-IR (TRIP 34938)* were obtained from Bloomington stock centre. UAS-*pickle* flies were obtained from NCBS *Drosophila* Resource Centre. *imd^{1}*, *spz^{rm7}*, *rel^{e20}*, *PGRP-LC^{E12}* and *PGRP-LE^{112}* flies were described previously (Neyen et al., 2014). Flies were maintained at 25°C and 65% humidity on a 12 hrs light/dark cycle, unless otherwise indicated. A full list of all genotypes used for each Figure can be found in Supplemental Table S2.

With the exception of *L.monocytogenes,* starter cultures of all bacterial cultures were initiated from single colonies grown on LB plates. Small volumes of the starter cultures were then diluted at least 1:1000 (so as to have an near undetectable OD) and cultured up to the desired OD on the day of the experiment. *Ecc15* was cultured in LB at 29°C with agitation to OD 0.3. *M.luteus* was cultured in LB at 37°C with agitation to OD 0.3. *P.ret* was cultured in LB at 37°C with agitation to OD 0.1 (low dose) or OD 1 (high dose). *B.subtilis* was cultured in LB at 37°C with agitation to OD 0.1. As an exception, *L.mono* was cultured overnight without agitation at 37°C in brain heart infusion (BHI) broth. The culture was initiated from a single colony grown on a BHI plate. The resulting overnight culture was then serially diluted to OD 0.2 using BHI broth.

Heat-killed (hk) bacteria were prepared as follows: *E.coli* (1106 strain) and *M.luteus* were cultured overnight in LB at 37°C with agitation. Cultures were initiated from single colonies grown on LB plates. After 24 hrs, cultures were spun down at 1600 rpm for 4 minutes at 4°C. Culture media was aspirated and remaining bacterial pellets were resuspended with sterile PBS to OD 1. Bacterial solutions were then heat-killed (hk) for 10 minutes at 95°C in a heating block. Heat-killed bacterial solutions were then prepared as followed: hk *M.lut*-only preparation = 9 volumes OD 1 *M.lut* (hk) suspension + 1 volume sterile PBS. *E.coli* (hk) only preparation = 1 volume OD 1 *E.coli* (hk) suspension + 9 volume sterile PBS. Mixtures of *M.lut* (hk) + *E.coli* (hk) preparation = 9 volumes OD 1 *M.lut* (hk) suspension + 1 volumes OD 1 *E.coli* (hk) suspension. These dilutions enabled the injection of approximately equal number of *E.coli* (hk) and *M.lut* (hk) respectively (live *E.coli* OD 1 contains approximately ten times as many CFUs as live *M.lut* OD 1). Preparations were then aliquoted and frozen at -80°C for repeat usage of identical hk bacterial preparations.

Oral infection and bleomycin treatment.

5-7 days old female flies were collected and kept at 18°C for 3 days (Gal80^{TS} ON, Gal4 OFF), and then were incubated at 29°C for seven days to knock-down the target gene (Gal80^{TS} OFF, Gal4 ON, RNAi ON). Flies were starved for 2 hrs at 29 ^{o}C and then transferred to infection vials covered with a filter paper (Whatman^{TM}) and 150 µl of an infection solution (*Ecc15* at OD 100, or *P.e.* at OD 50 in 2.5 % sucrose). Flies were dissected as described previously (Buchon et al., 2009; Houtz and Buchon, 2014). For bleomycin treatment, 5-7 day old female flies were raised, starved and fed on a Whatman filter paper covered by 150 µl of a solution of 250 µg/ml bleomycin (Sigma) in 2.5 % sucrose.

Lifespan analysis.

5 virgins *5966::GS* homozygotes were crossed to 1 male homozygote *UAS-lacZ-IR*, *UAS-pickle(GD)-IR* or *UAS-pickle(KK)-IR* in fly vials. 10 crosses were set per genotype. Progeny of these crosses were collected for 5 days after the first fly hatched. Flies were then allowed to mate for 2 days. Male siblings were then separated (20 per vial). For RU486 food supplementation, 100 µl of a 5 mg/ml solution of RU486 or vehicle (ethanol 80%) was deposited on top of the food and dried for at least 16 hrs. This resulted in a 0.2 mg/ml concentration of RU486 in the food accessible to flies (determined using a dye control as previously described for drug treatments (Grover et al., 2009)).

RT-qPCR and primer sequences.

RNA was extracted from whole flies, dissected midguts or S2* cells using either Trizol, Qiazol, Direct-zol RNA Mini Kit (Zymo Research) or RNeasy Mini Kit (QIAGEN). For whole fly analysis in Figures 3, 4, S3; pools of 15 male + 15 female flies were analysed. For whole fly analysis in Figure 6, 7, S5 and S6; pools of 5 female flies per sample were analysed. cDNAs were synthesized using Quantitect Reverse transcription kit (QIAGEN), and quantitative PCR was performed using MESA Blue qPCR mastermix Plus for SYBR assays (Eurogentech). SYBR green analysis was performed on a 7900HT machine (Applied Biosystems). For midgut analysis, a pool of 15 to 20 dissected female midguts was collected into Trizol (Invitrogen) for RNA extraction. Reverse transcription (RT) reactions were performed using the qScript cDNA synthesis kit (Quanta). Quantitative PCR was performed with SsoAdvanced™ Universal SYBR® Green Supermix (Bio-Rad) using CFX96 Touch^{TM} Real-Time PCR Detection System (Bio-Rad).

RT-qPCR SYBR Primer list.			
**CG number**	**Name**	**Forward**	**Reverse**
CG7939	*rp49*	GACGCTTCAAGGGACAGTATCTG	AAACGCGGTTCTGCATGAG
CG7629	*Attacin D*	GTCACTAGGGTTCCTCAG	GCCGAAATCGGACTTG
CG12763	*Diptericin A*	GCTGCGCAATCGCTTCTACT	TGGTGGAGTGGGCTTCATG
CG10794	*Diptericin B*	AGCCTGAACCACTGGCATA	AGATCGAATCCTTGCTTTGG
CG1385	*Defensin*	GTTCTTCGTTCTCGTGG	CTTTGAACCCCTTGGC
CG10810	*Drosomycin*	CGTGAGAACCTTTTCCAATATGATG	TCCCAGGACCACCAGCAT
CG5118	*pickle*	AACGGAAGAAAAGGGATGTCACC	GTTTCGGCGTACAGGGGTTTTA
CG15678	*pirk*	GGCGTTCGTGTGATAG	CTCAATGCGGTACTCC
CG33542	*unpaired 3*	GCGGGGAGGATGTACC	GTCTTCATGGAATGAGCC

Tissue culture and treatments.

Drosophila S2* cells were a kind gift from Neal Silverman. S2* cells were cultured at 23°C in Schneider's *Drosophila* medium (Gibco) supplemented with 10% FBS, 60 µg/ml Penicillin and 100 µg/ml Streptomycin. For transfection experiments, cells were seeded at 1 x 10^6 cells/ml density 1 day prior to transfections. For IPs/western blots, calcium phosphate (Clontech, Palo Alto, CA) was used to transiently transfect 3 µg total plasmid DNA (1:2 bait to prey ratio for IPs) according to manufacturer instructions. For RT-qPCR experiments, Effectene (QIAGEN) was used to transiently transfect 0.4 µg total plasmid DNA (1:4 inducer to *gfp/pirk/pickle* ratio) according to manufacturer instructions. For RT-qPCR experiments only, 1 day following transfection, cells were split back to 1 x 10^6 cells/ml density. Copper sulphate-inducible pMT vectors (ThermoFisher) were used throughout. Cells were treated with 0.7 mM copper sulphate two days after transfection, and collected after 6 hrs. RNAi knockdown was performed using 20 µg dsRNA as described previously (http://www.flyrnai.org/DRSC-PRR.html). For RNAi experiments, cells were subsequently treated with 1 µg/ml DAP-PGN (isolated from *E.coli)* and collected after 4 hrs. In all tissue culture experiments, cells were treated with 1 µM 20-hydroxyecdysone (Sigma) for 24 hrs prior to collection.

Immunoprecipitation, nuclear/cytoplasmic fractionation and western blot analysis.

For immunoprecipitation, cells were lysed on ice in S(+M) lysis buffer containing: 20 mM Tris pH 8.0, 40 mM $Na_4P_2O_7$, 50 mM NaF, 5 mM $MgCl_2$, 100 µM Na_3VO_4, 10 mM EDTA, 1 % Triton X-100, 0.5 % NaDOC plus Halt protease + phosphatase inhibitors (ThermoFisher). Lysates were run through a column (ThermoFisher) and total cell extracts were collected. FLAG or V5-tagged proteins were purified using antibody-IgG-coupled agarose beads (Sigma Aldrich, GE Healthcare respectively). After 2 hrs incubation at 4°C, beads were washed three times in S(+M) wash buffer: 20 mM Tris pH 8.0, 40 mM $Na_4P_2O_7$, 50 mM NaF, 5 mM $MgCl_2$, 100 µM Na_3VO_4, 10 mM EDTA, 0.1 % Triton X-100. For nuclear/cytoplasmic fractionation, cells were lysed on ice in cytoplasmic extraction buffer containing: 50 mM Tris pH 7.5, 137 mM NaCl, 10 % glycerol, 0.5 % Triton X-100. Extracts were spun at 14,000 r.p.m for 15 minutes and supernatants collected (cytoplasmic fraction). The remaining nuclear pellet

was washed three times in cytoplasmic extraction buffer and subsequently lysed in nuclear extraction buffer: 50 mM Tris pH 7.5, 137 mM NaCl, 10 % glycerol, 0.5 % Triton X-100, 1.5% SDS. The nuclear extract was vortexed, boiled and purified through a column (ThermoFisher) to ensure complete lysis. The following antibodies were used for western blotting: α-FLAG M2 (Sigma), α-V5 (Serotec), α-HA (Roche), α-Relish (ABIN1111036, antibodies-online), α-dHDAC1 (gift from Lori Pile), α-Tubulin (T9026, Sigma) and α-Histone 3 (MBL). Signals were visualised via chemiluminescence (GE Healthcare or Biorad Clarity).

Sequence collection and phylogenetic analysis

We used 11 biochemically characterized IκB protein sequences from *Homo sapiens* and *Drosophila melanogaster* sources (IκBα_Hs-CAB65556, IκBβ_Hs-AAH15528, IκBε_Hs-NP_004547, Bcl3_Hs-NP_005169, IκBNS_Hs-Q8NI38, IκBζ_Hs-Q9BYH8, NF-κB1_Hs-AAA36361, NF-κB2_Hs-CAC08399, Cactus_Dme-AAA85908, Relish_Dme-AAB17264 and Pickle_Dme-AAF51401.2) as query sequences for constructing IκB dataset. We constructed the dataset by utilizing the same sequences as mentioned in our previous analysis (Basith et al., 2013). Furthermore, few updated IκB sequences along with Pickle and its homologs were included in our new dataset. The Pickle homolog sequences (57 initial sequences) were obtained through BLAST search. Most of the sequences were discarded based on the filtering criteria described previously. The final twelve Pickle homologs were included in our final dataset for phylogenetic reconstruction. Since the previous dataset was too large, we included only a maximum of five IκB representative sequences (only in the case of vertebrates) for each species in our dataset. All IκB sequences were imported into the Geneious Trial software v8.1.2 (Kearse et al., 2012). Multiple sequence alignment (MSA) was performed using the plugin MAFFT v7.017 (Katoh et al., 2002) implemented in Geneious software. The MSAs were manually inspected and a few particularly gap-rich positions, poorly aligned and divergent regions from the alignments were excluded prior to the phylogenetic analysis. The final dataset contained a total of 148 sequences (including outgroup) from 75 organisms that were subjected to phylogenetic tree reconstructions (17 IκBα, 5 IκBβ, 18 IκBε, 8 Bcl3, 7 IκBNS, 11 IκBζ, 11 Cactus, 25 Relish, 17 NF-κB1, 16 NF-κB2, and 12 Pickle sequences). The MSAs were utilized to construct two rooted and two unrooted phylogenetic trees by UPGMA and neighbor joining (NJ) methods in Geneious Trial software v8.1.2. The molecular distances between the aligned sequences were calculated using the Jukes-Cantor genetic distance model. All gaps and missing data in the alignments were accounted for by pairwise deletions. Branch points were tested for significance by bootstrapping with 1000 replications and the most probable consensus tree was calculated with a support threshold of 50% for whole IκB phylogeny (combined vertebrate and invertebrate phylogeny) and 60% for invertebrate phylogeny constructions.

Model construction.

The three-dimensional (3D) model of Pickle was built using ANK-N5C (PDB ID: 4O60) as template, which shares a sequence identity of 26.8%. The target-template sequence alignments were

performed using MUSCLE (Edgar, 2004). The model was built using Modeller 9v8 (Eswar et al., 2006). We constructed 3D models using a distance restraint algorithm based on the MSA of the target sequence with the template structure by applying the CHARMM force field (Jo et al., 2008). An optimization method, which involved conjugate gradients and MD-simulated annealing, was employed to minimize violations of spatial restraints. For model building, the default parameters included in the “automodel” class were used. A series of 20 models were built, from which the best final model was selected based on RFMQA score. The quality of the models was assessed using RFMQA (Manavalan et al., 2014).

References

Basith, S., Manavalan, B., Gosu, V., and Choi, S. (2013). Evolutionary, structural and functional interplay of the IkappaB family members. PloS one *8*, e54178.

Buchon, N., Broderick, N.A., Poidevin, M., Pradervand, S., and Lemaitre, B. (2009). Drosophila intestinal response to bacterial infection: activation of host defense and stem cell proliferation. Cell host & microbe *5*, 200-211.

Edgar, R.C. (2004). MUSCLE: multiple sequence alignment with high accuracy and high throughput. Nucleic Acids Res *32*, 1792-1797.

Eswar, N., Webb, B., Marti-Renom, M.A., Madhusudhan, M.S., Eramian, D., Shen, M.Y., Pieper, U., and Sali, A. (2006). Comparative protein structure modeling using Modeller. Curr Protoc Bioinformatics *Chapter 5*, Unit 5 6.

Grover, D., Ford, D., Brown, C., Hoe, N., Erdem, A., Tavaré, S., and Tower, J. (2009). Hydrogen Peroxide Stimulates Activity and Alters Behavior in <italic>Drosophila melanogaster</italic>. PloS one *4*, e7580.

Houtz, P.L., and Buchon, N. (2014). Methods to assess intestinal stem cell activity in response to microbes in Drosophila melanogaster. Methods in molecular biology (Clifton, N.J.) *1213*, 171-182.

Jo, S., Kim, T., Iyer, V.G., and Im, W. (2008). CHARMM-GUI: a web-based graphical user interface for CHARMM. J Comput Chem *29*, 1859-1865.

Katoh, K., Misawa, K., Kuma, K., and Miyata, T. (2002). MAFFT: a novel method for rapid multiple sequence alignment based on fast Fourier transform. Nucleic Acids Res *30*, 3059-3066.

Kearse, M., Moir, R., Wilson, A., Stones-Havas, S., Cheung, M., Sturrock, S., Buxton, S., Cooper, A., Markowitz, S., Duran, C., *et al.* (2012). Geneious Basic: an integrated and extendable desktop software platform for the organization and analysis of sequence data. Bioinformatics *28*, 1647-1649.

Lhocine, N., Ribeiro, P.S., Buchon, N., Wepf, A., Wilson, R., Tenev, T., Lemaitre, B., Gstaiger, M., Meier, P., and Leulier, F. (2008). PIMS modulates immune tolerance by negatively regulating Drosophila innate immune signaling. Cell host & microbe *4*, 147-158.

Manavalan, B., Lee, J., and Lee, J. (2014). Random forest-based protein model quality assessment (RFMQA) using structural features and potential energy terms. PloS one *9*, e106542.

Neyen, C., Bretscher, A.J., Binggeli, O., and Lemaitre, B. (2014). Methods to study Drosophila immunity. Methods *68*, 116-128.
